# Microtubule forces drive nuclear damage in *LMNA* cardiomyopathy

**DOI:** 10.1038/s44161-025-00727-w

**Published:** 2025-10-10

**Authors:** Daria Amiad Pavlov, Julie Heffler, Carmen Suay-Corredera, Mohammad Dehghany, Kaitlyn M. Shen, Noam Zuela-Sopilniak, Rani Randell, Keita Uchida, Rajan Jain, Vivek Shenoy, Jan Lammerding, Benjamin Prosser

**Affiliations:** 1 Department of Physiology, Pennsylvania Muscle Institute, Perelman School of Medicine, University of Pennsylvania, Philadelphia, PA USA; 2 Weill Institute for Cell and Molecular Biology & Meinig School of Biomedical Engineering, Cornell University, Ithaca, NY USA; 3 Department of Materials Science and Engineering, Center for Engineering Mechanobiology, University of Pennsylvania, Philadelphia, PA USA; 4 Departments of Medicine and Cell and Developmental Biology, Penn Cardiovascular Institute, Penn Epigenetics Institute, Perelman School of Medicine, University of Pennsylvania, Philadelphia, PA USA

**Keywords:** Cardiomyopathies, Cell biology

## Abstract

Nuclear homeostasis requires balanced forces between the cytoskeleton and the nucleus. Mutations in *LMNA*, which encodes lamin A/C, weaken the nuclear lamina, leading to nuclear damage and muscle disease. Disrupting the linker of nucleoskeleton and cytoskeleton (LINC) complex, which connects the cytoskeleton to the nucleus, may ameliorate *LMNA*-associated cardiomyopathy, yet the cardioprotective mechanism remains unclear. Here we developed an assay to quantify the coupling between cardiomyocyte contraction and nuclear deformation and interrogate its dependence on the nuclear lamina and LINC complex. The LINC complex was mostly dispensable for transferring contractile strain to the nucleus, and its disruption did not rescue elevated nuclear strain in lamin A/C-deficient cardiomyocytes. Instead, LINC complex disruption eliminated the microtubule cage encircling the nucleus. Microtubule disruption prevented nuclear damage and preserved cardiac function in lamin A/C deficiency. Computational modeling revealed that microtubule forces create local stress concentrations that damage lamin A/C-deficient nuclei. These findings identify microtubule-dependent force transmission as a pathological driver and therapeutic target for *LMNA* cardiomyopathy.

## Abstract (web summary)

Amiad Pavlov, Heffler, et al. demonstrate that stress transmitted to the cardiomyocyte nucleus by the microtubule cage drives *LMNA*-associated cardiomyopathy and may represent a promising therapeutic target.

## Main

Cardiac muscle endures extreme mechanical stress from continuous contraction–relaxation cycles, necessitating unique adaptations of the cytoskeleton and nucleus. In adult cardiomyocytes, the actin-myosin, microtubule (MT) and desmin intermediate filament networks couple to the nucleus via the linker of nucleoskeleton and cytoskeleton (LINC) complex that spans the nuclear envelope (NE). The LINC complex consists of nesprin proteins, which contain a conserved KASH domain that interacts with SUN proteins spanning the inner nuclear membrane, which, in turn, connect to the nuclear lamina. The lamina is a meshwork of A-type and B-type lamin filaments that structurally support the nucleus and spatially organize chromatin^1,2^. Nuclear homeostasis depends on balance of these cytoskeletal forces, nuclear resistance and their coupling through the LINC complex^3^.

This balance is critical in the heart, as mutations in lamins and NE components disproportionately affect striated muscle^4,5^. Specifically, mutations in *LMNA* (encoding lamin A/C) cause a heterogeneous group of diseases (‘laminopathies’) that often include cardiomyopathy^6–8^. The *LMNA* N195K variant is associated with dilated cardiomyopathy, and *Lmna*^N195K/N195K^ mice develop heart failure and die within 12 weeks of age^9^. There are no specific therapies for *LMNA* cardiomyopathy, and disease mechanisms remain unclear. A prevailing hypothesis implicates mechanical damage to the NE upon lamina compromise^10–14^.

Importantly, decoupling the nucleus from the cytoskeleton via LINC complex disruption preserves cardiac function and extends lifespan in *Lmna* cardiomyopathy mice^15,16^. However, the cytoskeletal sources of nuclear damage and protective mechanism of LINC complex disruption remain unclear^10,13,16^, partly due to a lack of tools to directly measure how cytoskeletal forces are transferred to the nucleus^4,5,13^. Adult cardiomyocyte nuclei are interiorly located, surrounded by myofibrils and a cage of perinuclear MTs, hindering direct mechanical probe access^17^.

Here we introduce an assay to quantify sarcomere–nuclear strain coupling in beating, adult cardiomyocytes. We use this to interrogate the role of the LINC complex and cytoskeleton in driving nuclear strain during cardiomyocyte contraction. Surprisingly, sarcomere–nuclear strain coupling largely persists upon LINC complex disruption^18^, which also does not reduce contraction-induced nuclear strain in *Lmna*-deficient cardiomyocytes. Instead, LINC complex disruption eliminates the perinuclear MT cage, reducing MT-associated forces and protecting nuclear tips from rupture. MT disruption prevents nuclear damage in both mouse and human lamin-deficient cardiomyocytes and in a computational model simulating force distribution and nuclear vulnerability. Finally, in vivo disruption of the MT network protects nuclei from damage, preserves cardiac function and extends survival upon cardiomyocyte-specific *Lmna* depletion, underscoring the pathological role of MT-associated forces on fragile nuclei in *Lmna* cardiomyopathy.

## Results

### Sarcomere–nuclear strain coupling in beating, adult cardiomyocytes

**Fig. 1:**
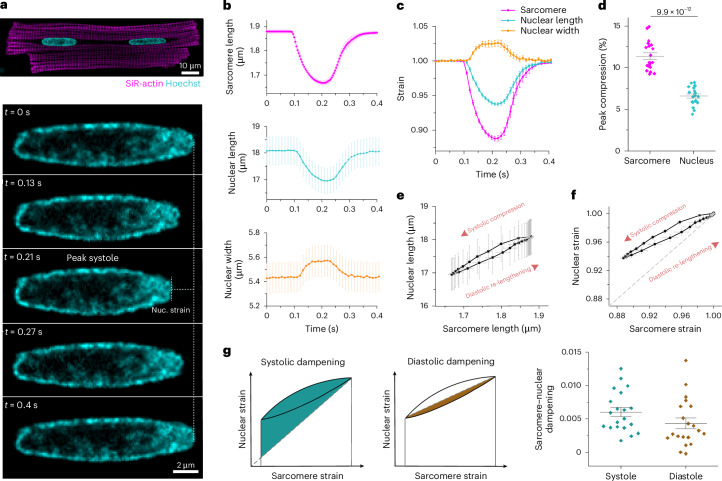
Active sarcomere–nuclear strain coupling in beating cardiomyocytes. **a**, Representative live isolated adult rat cardiomyocyte stained with SiR-actin and Hoechst 33342 to visualize sarcomeres and DNA, respectively (top). Cardiomyocytes were stimulated at 1 Hz, and the nucleus was imaged at 90 fps to follow nuclear deformation during the contraction cycle (bottom: representative time-lapse images; Supplementary Video 1). Nuc., nuclear. **b**, Sarcomere length, nuclear length and nuclear width recordings over time for a single contraction cycle. **c**, Sarcomere strain, nuclear length strain and nuclear width strain over time. **d**, Quantification of peak sarcomere and nuclear compression. **e**,**f**, Sarcomere–nuclear coupling represented with a plot of nuclear length versus sarcomere length (**e**) and respective nucleus strain versus sarcomere strain (**f**). The latter, dimensionless strain coupling map depicts the dampened strain on the nucleus during systolic compression and diastolic re-lengthening, as marked by the deviation from a linear correlation (dotted line). **g**, Sarcomere–nuclear strain dampening during systole quantified from area above linear correlation (left schematic). Diastolic dampening is quantified from area under end-systolic linear correlation (right schematic). Data are presented as mean ± s.e. for 20 cells from a single adult rat heart to illustrate the approach used throughout this study. Statistical significance was determined by two-tailed *t*-test.

Measuring active mechanical signal transfer into the nucleus is a missing component of interrogations into cardiac mechanobiology and *LMNA* cardiomyopathy. We developed a method to quantify sarcomere–nuclear strain coupling during electrically stimulated contractions in adult cardiomyocytes (Supplementary Video 1). We combined real-time measurements of sarcomere length change proximal to the nucleus with high-spatiotemporal-resolution imaging of nuclear deformation during stimulated contractions (Fig. 1a). Figure 1b demonstrates that, during the contraction cycle, sarcomere shortening and relaxation (magenta) are tightly coupled to the shortening and relaxation of nuclear length (cyan), with an opposing increase in nuclear width (orange). The relative change in sarcomere length (strain) during contraction is not fully transferred to the nucleus, resulting in dampening of nuclear strain (Fig. 1c). For example, for 11.4 ± 0.4% sarcomere compression at peak systole, nuclear length decreases only 6.6 ± 0.3% (Fig. 1d). We generated sarcomere–nuclear strain coupling maps by plotting absolute sarcomere length versus absolute nuclear length during systolic compression and diastolic re-lengthening (Fig. 1e) or the respective sarcomere strain versus nuclear strain (Fig. 1f; unless otherwise specified, ‘strain’ refers to strain along the contractile axis). The dotted line represents a theoretical scenario where sarcomere strain would be linearly elastically coupled, without loss, to nuclear strain. The upward deviation of the observed curve from the dotted line indicates dampening during contraction. We can quantify this sarcomere–nuclear strain dampening during both phases of the contractile cycle (Fig. 1g; see Methods for details).

**Fig. 2:**
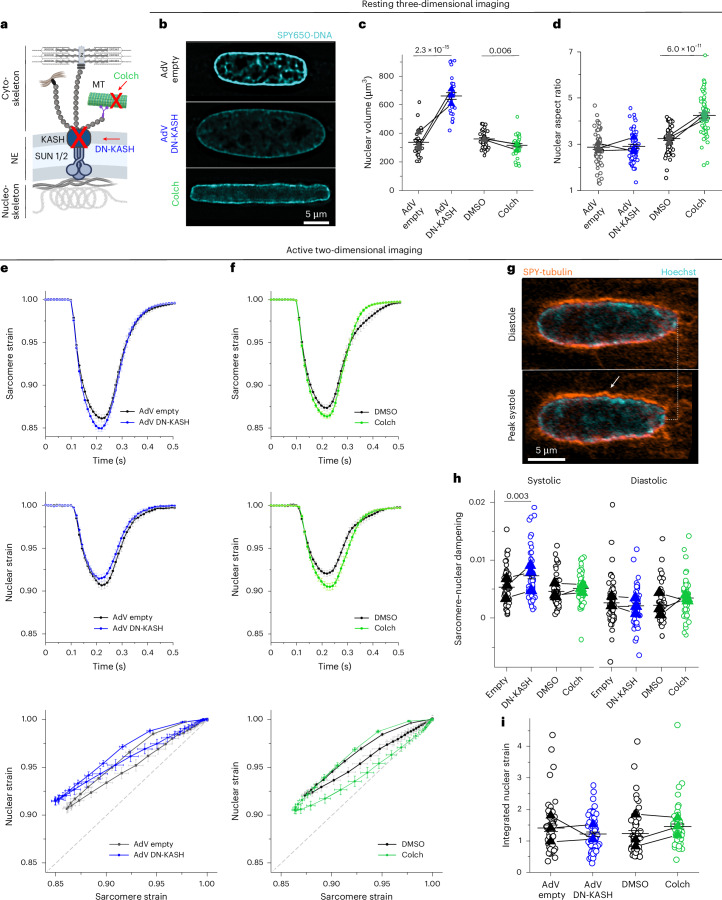
Distinct effects of LINC complex disruption and MT depolymerization on resting nuclear morphology and active sarcomere–nuclear strain coupling. **a**, Schematic of cytoskeletal-to-nucleoskeletal connections and the experimental perturbations. **b**, Live three-dimensional super-resolution imaging of resting adult rat cardiomyocyte nuclei (mid-nuclear planes are displayed). **c**,**d**, Nuclear volume (**c**) and aspect ratio (**d**) measurements after AdV DN-KASH or colchicine treatment. For **c**: AdV empty and AdV DN-KASH (48 hours): *N* = 3, *n* = 30, DMSO and colchicine (24 hours): *N* = 3, *n* = 36. For **d**: AdV empty and AdV DN-KASH (48 hours): *N* = 3, *n* = 51, DMSO and colchicine (24 hours): *N* = 3, *n* = 51. **e**,**f**, Active two-dimensional imaging of electrically stimulated cardiomyocytes. Sarcomere strain (top) and nuclear strain (middle) over time and sarcomere–nuclear strain coupling (bottom) for AdV DN-KASH (**e**) and colchicine (**f**) compared with their respective controls. **g**, Snapshots of live, WT cardiomyocyte labeled with SPY-555 tubulin and Hoechst 33342 during diastole and peak systole demonstrating MT cage buckling (arrow) during contraction. **h**, Quantification of sarcomere–nuclear strain dampening during the systolic and diastolic phases, for the indicated perturbations (see schematics in Fig. 1g). **i**, Integrated nuclear strain over time during the contractile cycle for the indicated perturbations. AdV empty and AdV DN-KASH (48 hours): *N* = 3, *n* = 51, DMSO and colchicine (24 hours): *N* = 3, *n* = 51. Data are presented as mean ± s.e. on individual cells. For **c**, **d**, **h** and **i**, individual cells are indicated with open circles, and individual animal replicate means are indicated with closed triangles connected by lines between experimental groups. Statistical significance was determined by two-tailed *t*-test. Colch, colchicine. Panel **a** created with BioRender.com.

**Extended Data Fig. 1:**
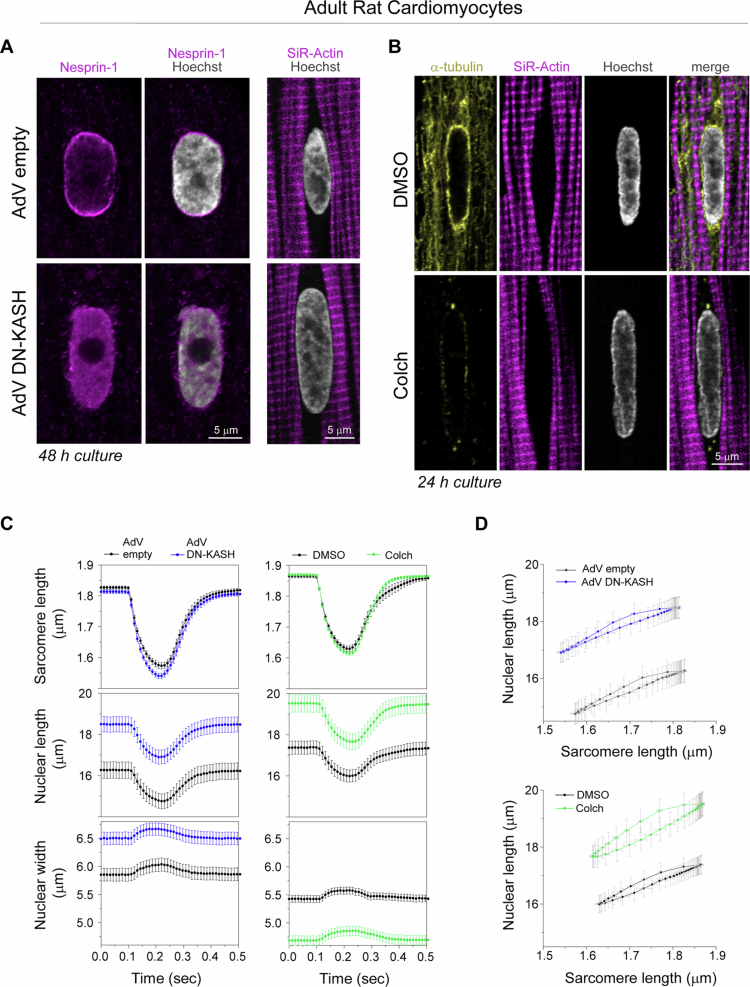
Validation of LINC complex and MT disruption in adult rat cardiomyocytes, and their effect on sarcomere–nuclear strain coupling. (**a**) Nesprin-1 perinuclear enrichment in adult rat cardiomyocyte is lost following 48 h of adenoviral DN-KASH transduction, with no apparent changes in perinuclear sarcomeric organization (labeled with SiR-actin). (**b**) MT network (yellow) is enriched at axial nuclear tips depleted of myofibrils (magenta). Colchicine treatment (1 µM) for 24 h results in complete loss of the MT network (including the dense perinuclear MT cage), with no apparent change in perinuclear sarcomeric organization (labeled with SiR-actin). Representative nuclear mid-plane images are shown. (**c**) Sarcomere length, nuclear length, and nuclear width recordings over time during stimulated cardiomyocyte contraction. Left - cardiomyocytes treated for 48 h in culture with AdV empty (black) or AdV DN-KASH (blue). Right - cardiomyocytes treated for 24 h in culture with DMSO (black) or 1 µM colchicine (green). (**d**) Nuclear length versus sarcomere length coupling plots for AdV DN-KASH and colchicine treatments. AdV empty and AdV DN-KASH (48 h): *N* = 3, *n* = 51. DMSO and colch (24 h): *N* = 3, *n* = 51. Data presented as mean ± SE.

We next interrogated cytoskeletal connections to the nucleus by either disrupting all cytoskeletal interactions with the LINC complex (AdV DN-KASH)^1,3^ or disrupting specifically MTs (1 µM colchicine; Fig. 2a). We confirmed that AdV DN-KASH disrupted the LINC complex 48 hours after AdV transduction through the loss of perinuclear nesprin-1 and that 24-hour colchicine resulted in the loss of MTs (Extended Data Fig. 1a,b). Neither acute LINC complex disruption nor MT depolymerization overtly altered sarcomere organization around the nucleus (Extended Data Fig. 1a,b) or resting sarcomere length (Extended Data Fig. 1c).

We assessed the effect of acute LINC complex and MT disruption on nuclear morphology via live, three-dimensional super-resolution imaging (Airyscan jDCV) of quiescent (non-contracting) adult rat cardiomyocytes (Fig. 2b). AdV DN-KASH increased nuclear size in all three dimensions, leading to a substantial increase in nuclear volume (Fig. 2c). Colchicine-treated nuclei displayed elongated morphology with increased nuclear aspect ratio and a subtle decrease in nuclear volume (Fig. 2c,d), in agreement with our previous report and consistent with reduced nuclear compression^3^.

We then assessed sarcomere–nuclear strain coupling in contracting cardiomyocytes. Upon electrical excitation, peak sarcomere contractility increased subtly with AdV DN-KASH, yet peak nuclear compression decreased slightly (Fig. 2e, top and middle, and Extended Data Fig. 1c, left). This decreased sarcomere–nuclear strain coupling is evident from the upward shift of the AdV DN-KASH curve (Fig. 2e, bottom) and from the significantly increased sarcomere–nuclear dampening during systole (Fig. 2h).

Colchicine treatment moderately increased sarcomere contraction and accelerated sarcomere relaxation (Fig. 2f, top), consistent with MTs providing a viscoelastic resistance to sarcomere motion^19^. Nuclear compression proportionately increased, but nuclear relaxation did not accelerate to match the faster sarcomere relaxation (Fig. 2f, top and middle). Faster sarcomere versus nuclear re-lengthening manifests as increased area, or hysteresis, within the diastolic strain coupling curve upon colchicine treatment (Fig. 2f, bottom). Buckling of the MT cage during systole (Fig. 2g and Supplementary Video 2) demonstrates the transmission of contractile forces to the nucleus through the MT cage. This buckling might provide restoring force to match sarcomere and nuclear relaxation rates during diastole^20^.

To examine the cumulative strain on the nucleus during the full contractile cycle, we integrated the nuclear strain over time and found no statistically significant differences between control versus LINC or MT disrupted cells (Fig. 2i). Together, these results indicate that nuclear strain during cardiomyocyte contraction is driven primarily by the shortening and re-lengthening of nearby sarcomeres, independent of their connectivity to the nucleus via the LINC complex.

### Cardiac-specific, in vivo LINC complex disruption protects from nuclear damage and cardiac dysfunction in *Lmna* N195K mice

**Fig. 3:**
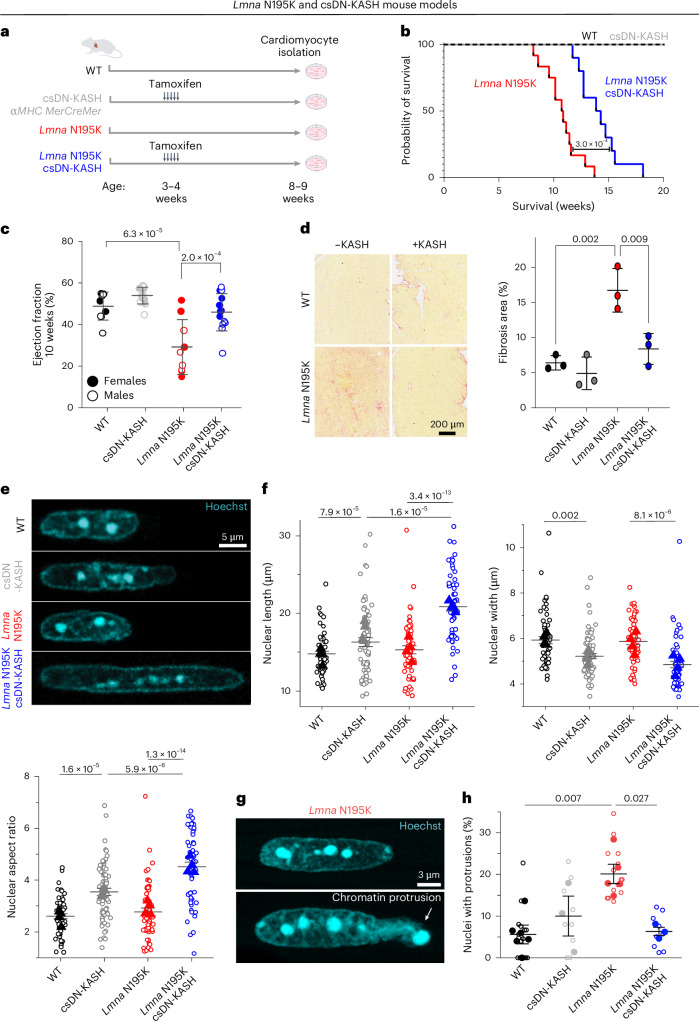
Cardiac-specific LINC complex disruption extends lifespan, improves cardiac function and protects against nuclear ruptures in *Lmna* N195K cardiomyopathy. **a**, Mouse models and experimental timelines used in this study. **b**, Kaplan–Meier survival plot. *N* = 10 mice per genotype. **c**, Left ventricular ejection fraction measured by echocardiography at 10 weeks of age. Closed circles denote females; open circles denote males. WT: *N* = 9, csDN-KASH: *N* = 15, *Lmna* N195K: *N* = 9, *Lmna* N195K csDN-KASH: *N* = 13. Error bar represents mean ± s.d. Statistical significance was determined by one-way ANOVA with Tukeyʼs multiple correction. **d**, Representative Picrosirius red–stained heart sections and quantification of % fibrotic area. *N* = 3 animal replicates per group. Error bar represents mean ± s.d. Statistical significance was determined by one-way ANOVA with Tukeyʼs multiple correction. **e**, Representative images of cardiomyocyte nuclear morphology at 8–9 weeks of age. **f**, Quantification of nuclear length, width and aspect ratio. WT: *N* = 4, *n* = 58. csDN-KASH: *N* = 4, *n* = 69. *Lmna* N195K: *N* = 4, *n* = 55. *Lmna* N195K csDN-KASH: *N* = 4, *n* = 59. Data are presented as mean ± s.e. of individual nuclei (open circles); individual animal replicate means are indicated with closed triangles. **g**, Chromatin protrusion from the nucleus in a *Lmna* N195K cardiomyocyte. Representative mid-plane images of a normal *Lmna* N195K nucleus (top) and a nucleus with a chromatin protrusion (bottom) in *Lmna* N195K cardiomyocytes. **h**, Quantification of the percentage of nuclei with chromatin protrusions in each genotype by three independent, blinded scorers. Open circles represent the percentage of nuclei with chromatin protrusions quantified by each independent user for each animal; closed circles correspond to the mean for each animal. Mean ± s.e. is shown for each genotype. WT: *N* = 5, *n* = 223. csDN-KASH: *N* = 3, *n* = 75. *Lmna* N195K: *N* = 5, *n* = 287. *Lmna* N195K csDN-KASH: *N* = 3, *n* = 223. Statistical significance was determined by one-way ANOVA with Bonferroni correction. Panel **a** created with BioRender.com.

**Extended Data Fig. 2:**
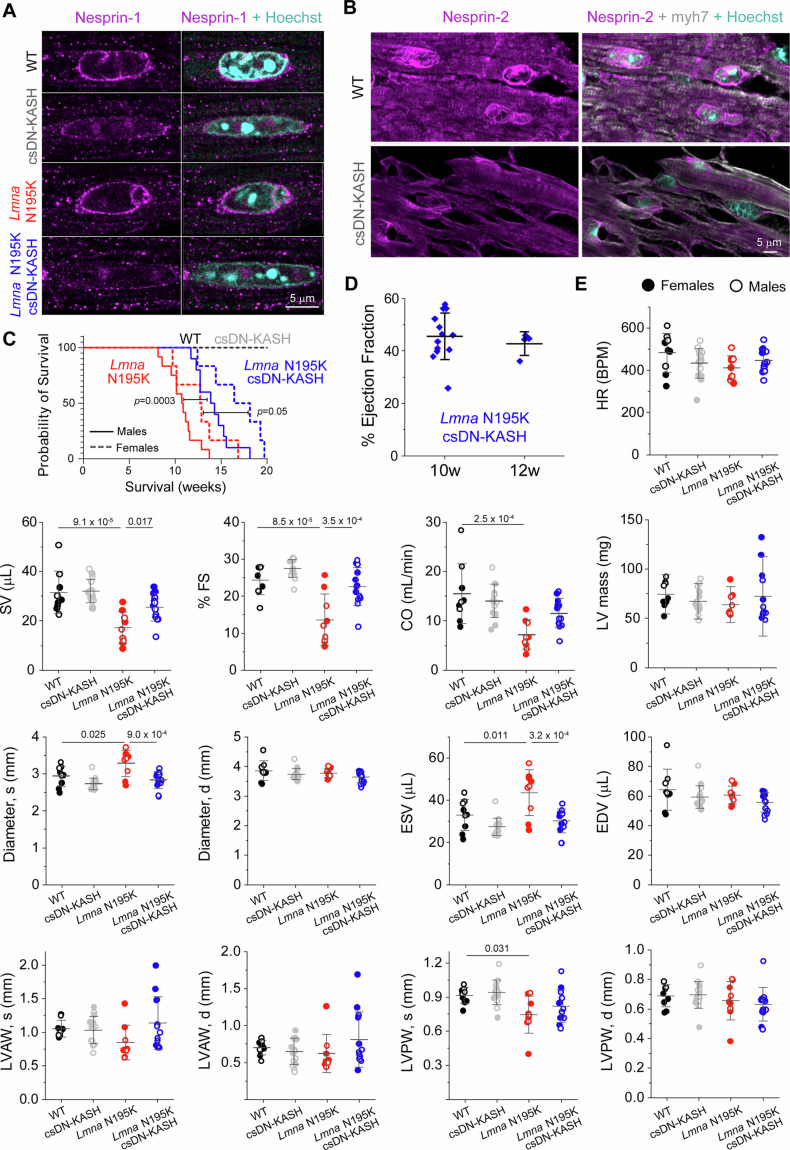
Validation of inducible, cardiac specific LINC complex disruption in WT and Lmna N195K mice. (**a**) Nesprin-1 perinuclear enrichment in adult WT and *Lmna* N195K mouse cardiomyocytes is lost following cardiac specific in-vivo disruption of the LINC complex (csDN-KASH). Representative nuclear mid-plane images are shown. (**b**) Nesprin-2 perinuclear enrichment in cardiomyocytes is lost in csDN-KASH mice (left panel). Representative tissue sections are shown. Cardiomyocyte nuclei identified by myh7 and Hoechst counterstain (right panel) (**c**) Kaplan-Meier survival plot of the different experimental groups separated to males (solid line) and females (dashed line). (**d**) Left ventricular ejection fraction measured by echocardiography, for the *Lmna* N195K csDN-KASH mice at 10 and 12 weeks of age. 10 weeks: *N* = 13, 12 weeks: *N* = 4. Error bars represent mean ± SD. (**e**) Echocardiography measurements at 10 weeks of age for the indicated groups. Heart rate (HR), stroke volume (SV), % fractional shortening (FS), cardiac output (CO), corrected left-ventricular mass (LV mass), end-systolic and end-diastolic left ventricular diameters (Diameter, s and d), end-systolic and end-diastolic volume (ESV, EDV), end-systolic and end-diastolic left-ventricle anterior and posterior wall thickness (LVAW, s and d, LVPW s and d). Closed circles denote females, open circles denote males. WT: *N* = 9, csDN-KASH: *N* = 15, *Lmna* N195K: *N* = 9, *Lmna* N195K csDN-KASH *N* = 13. Error bar represents mean ± SD. Statistical significance determined by one-way ANOVA with Tukey multiple correction.

We next sought to investigate the role of cytoskeletal forces in nuclear damage driven by lamin A/C deficiency or disease-causing *Lmna* mutations. We used the previously described *Lmna*^N195K/N195K^ (hereafter ‘*Lmna* N195K’) mutant mouse model with established dilated cardiomyopathy (DCM) phenotype^9^ and mechanical instability of the nucleus, similar to that observed with complete deletion of lamin A/C^10,11^. Recent studies found that global and cardiac-specific LINC complex disruption improves cardiac function and prolongs lifespan in *Lmna* N195K and other *Lmna* mutant mice^15,16^. To probe the cardiomyocyte-specific effects of LINC complex disruption, we used the previously described inducible *αMHC-MerCreMer* DN-KASH mouse model (referred to as cardiac-specific DN-KASH, or csDN-KASH)^18^. We crossed these mice to *Lmna* N195K mice to generate a *Lmna* cardiomyopathy model with cardiac-specific inducible LINC complex disruption. LINC complex disruption was induced by five consecutive daily tamoxifen injections at 3–4 weeks of age, followed by cardiomyocyte isolation at 8–9 weeks of age, allowing for 5 weeks of in vivo LINC complex disruption (Fig. 3a). We confirmed LINC complex disruption upon csDN-KASH induction in cardiomyocytes by the loss of perinuclear nesprin-1 and nesprin-2 (Extended Data Fig. 2a,b). Consistent with previous reports^15,16^, LINC complex disruption significantly extended the lifespan of *Lmna* N195K mice (Fig. 3b and separated by gender in Extended Data Fig. 2c), improved cardiac contractility (Fig. 3c) and structure (Extended Data Fig. 2e) and reduced cardiac fibrosis (Fig. 3d). Cardiac function was largely maintained in *Lmna* N195K csDN-KASH mice even at 12 weeks of age, when most of the *Lmna* N195K controls without LINC complex disruption had died (Extended Data Fig. 2d).

**Extended Data Fig. 3:**
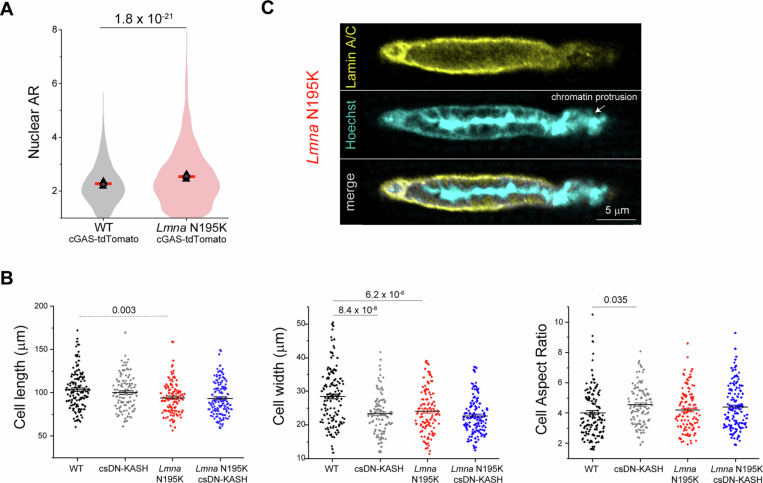
Nuclear and cardiomyocyte morphology in Lmna N195K mice. (**a**) Quantification of nuclear aspect ratio in cardiomyocytes isolated from WT and *Lmna* N195K mice carrying cGAS-tdTomato reporter. Red lines represent mean of pooled nuclei, and black triangles correspond to individual replicate means. WT cGAS-tdTomato: *N* = 4, *n* = 1898. *Lmna* N195K cGAS-tdTomato: *N* = 4, *n* = 2915. (**b**) Isolated cardiomyocyte cell length, width and cell aspect ratio for the indicated mouse models. WT: *N* = 3, *n* = 133. csDN-KASH: *N* = 3, *n* = 105. *Lmna* N195K: *N* = 3, *n* = 113. *Lmna* N195K csDN-KASH: *N* = 3, *n* = 143. Data presented as mean ± SE. Statistical significance determined by 1-way ANOVA with Bonferroni correction. (**c**) NE rupture in *Lmna* N195K cardiomyocytes manifested by chromatin protrusion from the nucleus, with partial lamina coverage. Representative mid-plane confocal image of the nucleus is shown to illustrate ruptured lamina. Protrusions quantified from full data set in main Fig. 3h.

We did not observe significant changes in nuclear dimensions between wild-type (WT) and *Lmna* N195K cardiomyocytes using high-resolution imaging (Fig. 3e,f). A separate analysis of a larger, albeit lower-resolution, dataset revealed a subtle (11%) but statistically significant increase in nuclear aspect ratio in *Lmna* N195K cardiomyocytes (Extended Data Fig. 3a). By contrast, LINC complex disruption (csDN-KASH) resulted in substantial elongation of WT cardiomyocyte nuclei (36% increase in nuclear aspect ratio) and even more extreme nuclear elongation in *Lmna* N195K csDN-KASH mice (63% increase in nuclear aspect ratio; Fig. 3e,f). This nuclear elongation occurred independent of any corresponding changes in cellular length, width or aspect ratio (Extended Data Fig. 3b), instead indicating an altered balance between cytoskeletal forces acting on the nucleus and internal nuclear resistance^3^. Notably, the increased nuclear aspect ratio due to in vivo LINC complex disruption mirrored the alterations in nuclear morphology upon colchicine treatment (Figs. 2d and 3f), suggesting that it may arise from a reduction in MT compressive forces.

We observed increased nuclear fragility and NE rupture in *Lmna* N195K versus WT nuclei, scored as chromatin spilling out of the nucleus (Fig. 3g) with the nuclear lamina only partially enclosing the protrusion (Extended data Fig. 3c). In agreement with recent findings in *Lmna* knockout mice^14^, we observed chromatin protrusions in *Lmna* N195K cardiomyocytes exclusively at the tips of nuclei. Using blinded scoring, we identified chromatin protrusions in 20% of nuclei from *Lmna* N195K cardiomyocytes (Fig. 3h; see Methods for details on scoring criteria). Notably, in vivo LINC complex disruption reduced NE ruptures in *Lmna* N195K mice back to levels of WT controls (Fig. 3h). Taken together, these findings confirm the protective effect of LINC complex disruption on NE ruptures, lifespan and cardiac function in *Lmna* N195K mice.

### In vivo LINC complex disruption does not reduce nuclear strain during myocyte contraction

**Fig. 4:**
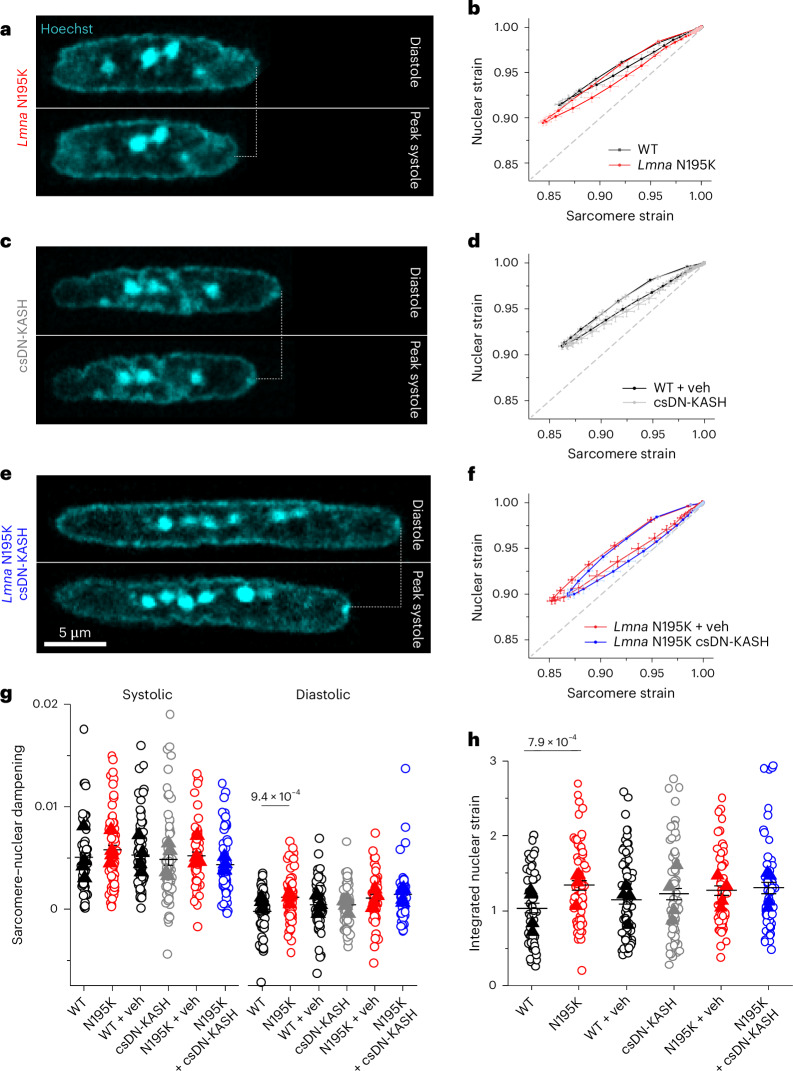
Increased active nuclear strain in *Lmna* cardiomyopathy is not restored by cardiac-specific in vivo LINC complex disruption. **a**, Representative snapshots of *Lmna* N195K cardiomyocyte nuclei during diastole and peak systole. **b**, Increased nuclear compression as evidenced by a downward shift in the laminopathy strain coupling curve. **c**,**d**, Representative snapshots of cardiac-specific LINC complex disruption in WT cardiomyocyte nuclei during diastole and peak systole (**c**) with no change in active strain coupling (**d**). **e**,**f**, Representative snapshots of cardiac-specific LINC complex disruption in *Lmna* N195K cardiomyocyte nuclei during diastole and peak systole (**e**) with no change in active strain coupling (**f**). Data are presented as mean ± s.e. **g**, Quantification of sarcomere–nuclear strain dampening during the systolic and diastolic phases, for the indicated groups (see schematics in Fig. 1g). **h**, Integrated nuclear strain over time during the contractile cycle for the indicated groups. WT: *N* = 4, *n* = 58. *Lmna* N195K: *N* = 4, *n* = 69. WT veh: *N* = 4, *n* = 81. csDN-KASH: *N* = 4, *n* = 69. *Lmna* N195K veh: *N* = 4, *n* = 55. *Lmna* N195K csDN-KASH: *N* = 4, *n* = 59. Data are presented as mean ± s.e. of individual cells (open circles); individual animal replicate means are indicated with closed triangles. Statistical significance was determined by two-tailed *t*-test. veh, vehicle.

**Extended Data Fig. 4:**
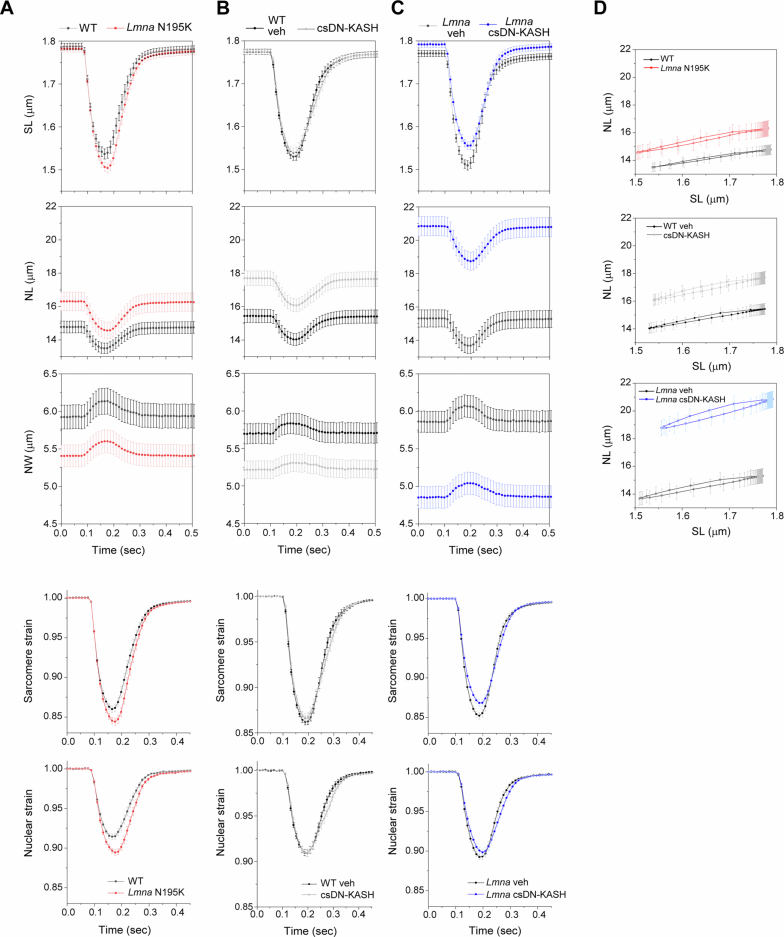
Active sarcomere–nuclear coupling for cardiac specific LINC complex disruption in WT and Lmna N195K mice. Sarcomere length (SL), nuclear length (NL), and nuclear width (NW) recordings over time during stimulated contraction of freshly isolated cardiomyocytes 8-9 weeks of age. Sarcomere strain and nuclear strain over time are plotted below. (**a**) *Lmna* N195K (red) and WT littermate controls (black). (**b**) Cardiac specific LINC complex disruption (gray) and WT vehicle controls (black). (**c**) *Lmna* N195K with cardiac specific LINC complex disruption (blue) and littermate *Lmna* N195K controls (black). (**d**) NL versus SL coupling plots for the corresponding experimental groups. WT: *N* = 4, *n* = 58. *Lmna* N195K: *N* = 4, *n* = 69. WT veh: *N* = 4, *n* = 81. csDN-KASH: *N* = 4, *n* = 69. *Lmna* N195K veh: *N* = 4, *n* = 55. *Lmna* N195K csDN-KASH: *N* = 4, *n* = 59. Data presented as mean ± SE.

A prevailing hypothesis for NE ruptures in laminopathies is that fragile nuclei are more susceptible to mechanical damage induced by forceful sarcomeric contractions^13^. Therefore, decoupling the nucleus from the cytoskeleton might reduce contractility-induced nuclear damage^15^. To probe this hypothesis, we first asked whether *Lmna* N195K cardiomyocyte nuclei indeed undergo more strain during the contractile cycle. Consistent with a more deformable nucleus^10^, *Lmna* N195K cardiomyocytes showed increased nuclear compression during sarcomere contraction (Fig. 4a and Extended Data Fig. 4a) and nuclear re-lengthening that lagged behind sarcomere re-lengthening. This resulted in a downward shift in the strain coupling curve (Fig. 4b), increased diastolic sarcomere–nuclear dampening (Fig. 4g) and a significant increase in integrated nuclear strain over the contractile cycle in *Lmna* N195K cardiomyocytes (Fig. 4h). These data provide, to our knowledge, the first direct evidence for increased nuclear strain in mature, contracting mutant *Lmna* cardiomyocytes.

We hypothesized that LINC complex disruption may normalize the increased nuclear strain in *Lmna* N195K cardiomyocytes. Surprisingly, nuclear compression was not reduced in csDN-KASH cardiomyocytes (Fig. 4c,e and Extended Data Fig. 4b), and the overall strain coupling curve (Fig. 4d) and sarcomere–nuclear dampening (Fig. 4g) remained unchanged. These data further indicate that an intact LINC complex is not required for transferring the majority of sarcomeric strain into the nucleus during myocyte contraction. Consistently, neither nuclear compression (Extended Data Fig. 4c) nor sarcomere–nuclear dampening (Fig. 4g) nor active strain coupling (Fig. 4f) were altered in *Lmna* N195K csDN-KASH myocytes compared with *Lmna* N195K controls. The increased integrated nuclear strain in *Lmna* N195K mutants was not rescued by in vivo LINC complex disruption (Fig. 4h). These results indicate that, although more deformable *Lmna* N195K nuclei undergo increased strain during the contractile cycle, this effect is not rescued by LINC complex disruption, suggesting that cardioprotection is conferred through an alternative mechanism.

### In vivo LINC complex disruption eliminates the perinuclear MT cage

**Fig. 5:**
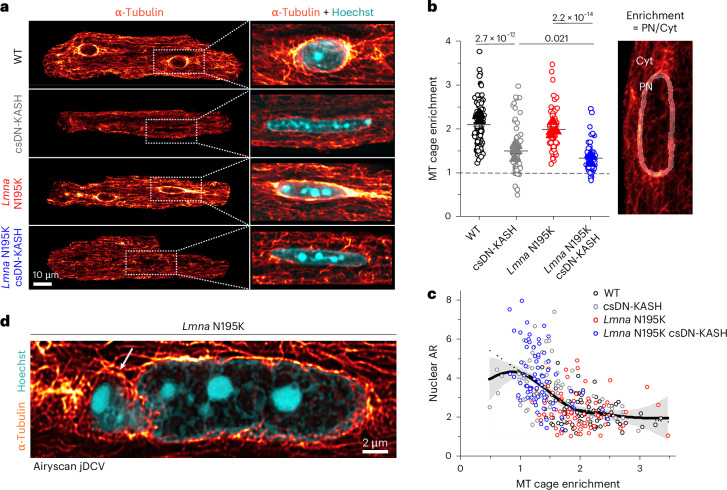
In vivo LINC complex disruption eliminates perinuclear MT cage, leading to nuclear elongation. **a**, Representative mid-plane immunofluorescence images of the MT network (α-tubulin), with a zoom-in on a merge with labeled nuclei (Hoechst) in the WT, csDN-KASH, *Lmna* N195K and *Lmna* N195K csDN-KASH adult mouse cardiomyocytes. **b**, Quantification of perinuclear MT enrichment defined as perinuclear (PN) to cytoplasmic (Cyt) α-tubulin ratio (illustrated on the image on the right). Data are presented as mean ± s.e. of individual nuclei (open circles); individual animal replicate means are indicated with closed triangles. Statistical significance was determined by one-way ANOVA with Bonferroni correction. **c**, Nuclear aspect ratio (AR) as a function of PN MT enrichment. Open circles represent individual nuclei from the respective groups. A fit to the pooled data is depicted with continuous black line. Gray area represents the error to the fit. Biphasic piecewise linear regression fit is indicated with a dashed black line (see Methods for details). **d**, Super-resolution Airyscan jDCV mid-plane image of a *Lmna* N195K cardiomyocyte nucleus (Hoechst) with the dense MT network (α-tubulin) penetrating the chromatin protrusion (white arrow). WT: *N* = 3, *n* = 85. csDN-KASH: *N* = 3, *n* = 71. *Lmna* N195K: *N* = 3, *n* = 70. *Lmna* N195K csDN-KASH: *N* = 3, *n* = 75.

**Extended Data Fig. 5:**
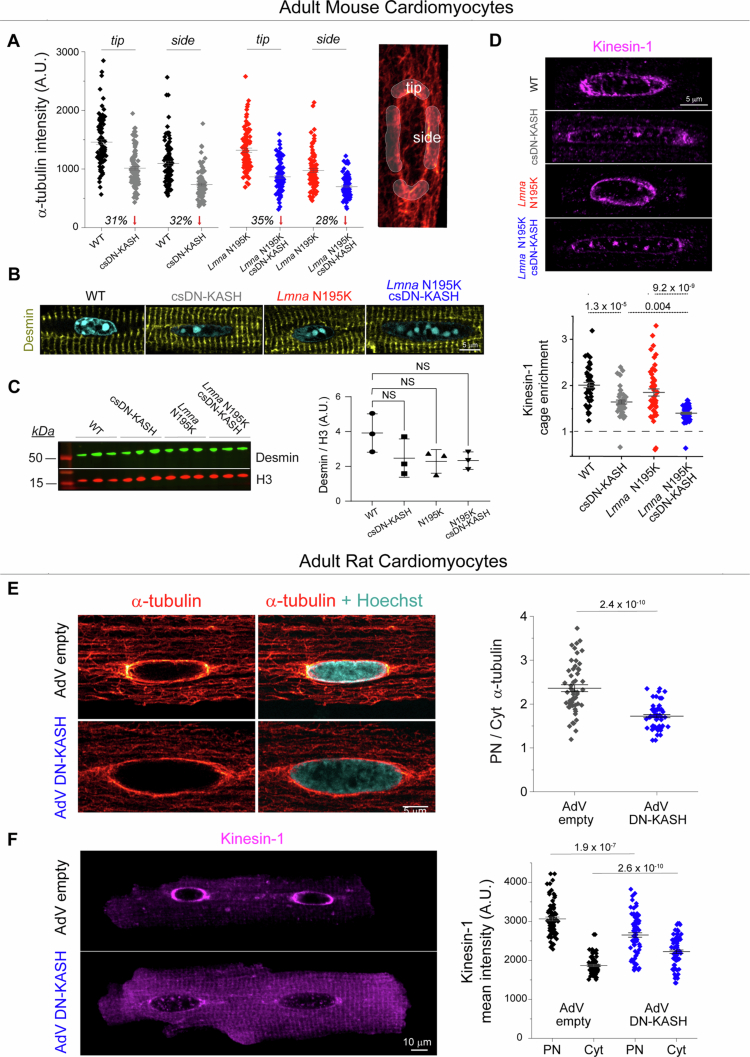
Cytoskeletal characterization of rat and mouse cardiomyocytes with LINC complex disruption. (**a**) Quantification of α-tubulin enrichment at the nuclear tips and sides in the indicated mouse model cardiomyocytes. (**b**) Representative mid-plane immunofluorescence images of desmin intermediate filament network surrounding the nuclei (Hoechst) of the indicated groups. (**c**) Desmin western blot and quantification, normalized to H3, for the indicated groups. (**d**) Representative mid-plane immunofluorescence images of kinesin-1 and quantification of perinuclear kinesin-1 enrichment, defined as PN to Cyt kinesin-1 ratio. WT: *N* = 2, *n* = 46. csDN-KASH: *N* = 2, *n* = 51. *Lmna* N195K: *N* = 2, *n* = 50. *Lmna* N195K csDN-KASH: *N* = 2, *n* = 59. (**e**) Representative nuclear mid-plane images of adult rat cardiomyocytes labeled with α-tubulin and Hoechst (left), following 48 h of adenoviral LINC complex disruption. Quantification of perinuclear α-tubulin enrichment is shown on the right. AdV empty and AdV DN-KASH: *N* = 2, *n* = 29. (**f**) Representative nuclear mid-plane images of kinesin-1 in the entire adult rat cardiomyocyte following 48 h of adenoviral LINC complex disruption (left). Quantification of kinesin-1 intensity separately at the perinucleus and the cytoplasm is shown on the right. AdV empty: *N* = 2, *n* = 78, AdV DN-KASH: *N* = 2, *n* = 80. Data presented as mean ± SE. Statistical significance determined by two-tailed t-test. Source data

We next explored alternative sources of mechanical stress that could damage *Lmna*-deficient nuclei and be reduced by LINC complex disruption. We hypothesized that nuclear elongation upon csDN-KASH might be a consequence of reduced MT compression on the nucleus. Consistently, we observed almost complete elimination of the perinuclear MT cage upon csDN-KASH induction in WT and *Lmna* N195K cardiomyocytes (Fig. 5a,b). The perinuclear MT cage in cardiomyocytes was specifically enriched at the nuclear tips (Extended Data Fig. 5a), which might contribute to increased mechanical forces at this location where chromatin protrusions were exclusively observed (Fig. 3g and Extended Data Fig. 3c).

We leveraged intracellular heterogeneity to assess the dependence of nuclear aspect ratios on levels of perinuclear MT enrichment, where we observed a biphasic relationship (Fig. 5c). A fit to the pooled data (Fig. 5c, continuous black trace with gray error area; see Methods for details) revealed a deflection point at ‘MT cage enrichment’ = 1.9. A piecewise linear regression (dashed black trace) indicates a negative correlation between nuclear aspect ratio and MT cage enrichment only below the deflection point. Together, these observations support the hypothesis that nuclear elongation upon in vivo LINC complex disruption is likely driven by decoupling of the MT network from the nucleus, with even greater elongation in more deformable *Lmna* N195K nuclei.

We, thus, hypothesized that nuclear damage in *Lmna* N195K nuclei may be driven by perinuclear MT-dependent forces. In support, super-resolution images in *Lmna* N195K cardiomyocytes showed MTs encaging the protruded chromatin at nuclear tips (Fig. 5d). Desmin intermediate filaments help balance compressive MT forces on the nucleus in mature cardiomyocytes^3^; thus, desmin deficiency could potentially contribute to such nuclear damage. However, we did not observe altered desmin levels or organization between groups (Extended Data Fig. 5b,c), suggesting that nuclear fragility, and not desmin deficiency, facilitates MT-based nuclear damage in *Lmna* N195K cardiomyocytes.

MTs interact with the LINC complex via kinesin motors, and nesprins are required to recruit kinesin motors and MTs to the nuclear periphery^21^. We found that LINC complex disruption depletes kinesin-1 from the perinuclear space (Extended Data Fig. 5d), in agreement with previous reports^10,16,22^. We also observed robust perinuclear kinesin-1 depletion with partial loss of the perinuclear MT cage after 48-hour AdV DN-KASH transduction in isolated adult rat cardiomyocytes (Extended Data Fig. 5e,f). These findings suggest that LINC complex disruption leads to rapid loss of perinuclear kinesin motors and eventual loss of the perinuclear MT cage.

### *Lmna* N195K mutant and lamin A/C-deficient cardiomyocytes have increased NE ruptures linked to perinuclear MTs

**Fig. 6:**
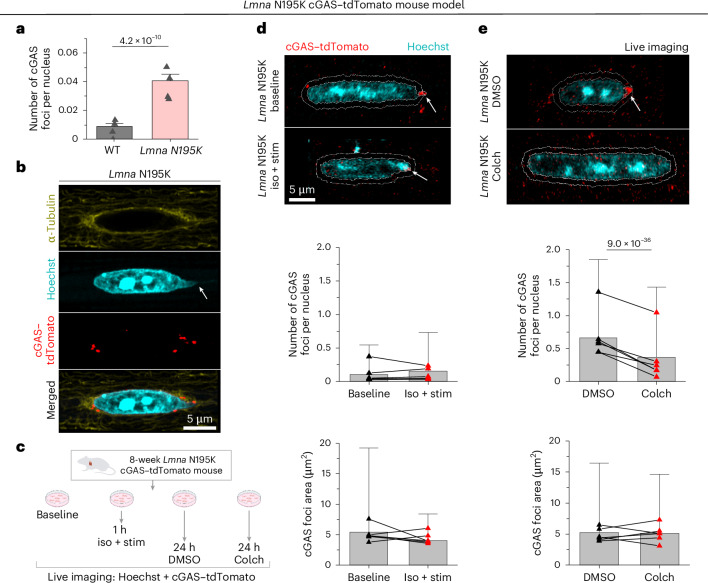
MT disruption protects from nuclear damage in *Lmna* N195K cardiomyocytes. **a**, Quantification of number of cGAS foci per nucleus for WT (*N* = 4, *n* = 1,898) and *Lmna* N195K (*N* = 4, *n* = 2,915) groups. Bar graphs represent the mean ± 1 s.d. of the pooled nuclei. Superimposed black triangles represent the replicate means. Statistical significance was determined by two-tailed *t*-test. **b**, Immunofluorescent mid-plane image of the MT network (α-tubulin) on the tip of *Lmna* N195K nucleus, associated with a chromatin protrusion (Hoechst, arrow) and cGAS foci (cGAS–tdTomato) indicative of NE ruptures. **c**, Experimental design for live imaging of perinuclear cGAS–tdTomato foci in a *Lmna* N195K mouse model, after in vitro stimulation in the presence of isoproterenol or colchicine treatment. **d**,**e**, Representative MIP images of nuclei (Hoechst) and perinuclear cGAS–tdTomato foci, from freshly isolated (baseline) and contractility-induced (iso + stim) cardiomyocytes (**d**) and DMSO-treated or colchicine-treated cardiomyocytes (**e**). The 1-µm perinuclear rings used for foci identification are indicated with white lines; the detected cGAS foci are indicated with arrows. Quantification of the number and area of perinuclear cGAS foci, per nucleus (bottom). *N* = 6, *n* = 4,869 (baseline), *n* = 2,286 (1 hour iso + stim), *n* = 4,820 (24 hour DMSO), *n* = 4,263 (24 hour colchicine). Bar graphs represent the mean ± 1 s.d. of the pooled nuclei. Superimposed black triangles represent the replicate means, connected by lines to their respective treatment conditions. Statistical significance was determined by two-tailed *t*-test. Iso, isoproterenol; stim, stimulation. Panel **c** created with BioRender.com.

**Extended Data Fig. 6:**
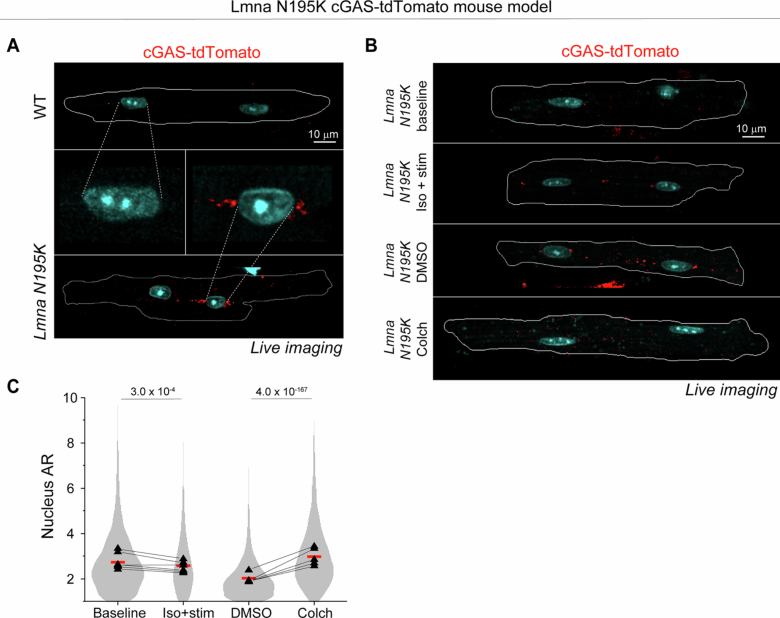
Full cardiomyocyte views and nuclear aspect ratio quantification for Lmna N195K cGAS-tdTomato experimental groups. (**a**) Full cell views of representative maximum intensity projection images labeled for nuclei (Hoechst) and cGAS-tdTomato (red), from freshly isolated cGAS-tdTomato WT, and cGAS-tdTomato *Lmna* N195K mouse models. The middle panel shows a zoom in on the nucleus and the perinuclear region used for cGAS foci quantification. Relates to the full data set quantified in main Fig. 6a. (**b**) Full cell views of representative maximum intensity projection images labeled for nuclei (Hoechst) and cGAS-tdTomato (red) for the indicated experimental groups. (**c**) Nuclear aspect ratio (AR) for the experimental groups of cGAS-tdTomato *Lmna* N195K model. *N* = 6, *n* = 4869 (baseline), *n* = 2286 (1 h iso + stim), *n* = 4820 (24 h DMSO), *n* = 4263 (24 h colch). Red lines represent the puled nuclei mean for each experimental group. Superimposed black triangles represent the replicate means and connected by lines to their respective treatment conditions. Statistical significance determined by 1-way ANOVA with Bonferroni correction.

To directly test the role of MTs in nuclear ruptures, we used transgenic mice expressing a fluorescent cGAS–tdTomato reporter^10^ crossed with the *Lmna* N195K mouse model to allow quantification of NE rupture in live cardiomyocytes. cGAS–tdTomato foci were observed in and around the nucleus in the *Lmna* N195K group and were significantly increased compared with cGAS–tdTomato WT mice (Fig. 6a and Extended Data Fig. 6a). In *Lmna* N195K cardiomyocytes, cGAS foci were mostly observed at the nuclear tips, regardless of the absence or presence of chromatin protrusions (Fig. 6b).

We asked whether increased contractile workload and/or MT disruption would alter NE ruptures in cardiomyocytes isolated from cGAS–tdTomato *Lmna* N195K mice (Fig. 6c). Augmented systolic workload induced by 1-hour, 0.5-Hz electrical stimulation and isoproterenol affected neither the number nor the size of perinuclear cGAS–tdTomato foci (Fig. 6d), indicating that, over this duration, active sarcomere contractility did not promote NE rupture. Alternatively, 24 hours of MT disruption (1 µM colchicine) induced nuclear elongation, consistent with reduced compressive forces (Extended Data Fig. 6c), and decreased the number of perinuclear cGAS–tdTomato foci, with no change in their size (Fig. 6e). These data suggest that MT disruption prevents the formation of new NE ruptures in *Lmna* N195K cardiomyocytes, without modulating the size of pre-existing ruptures.

**Extended Data Fig. 7:**
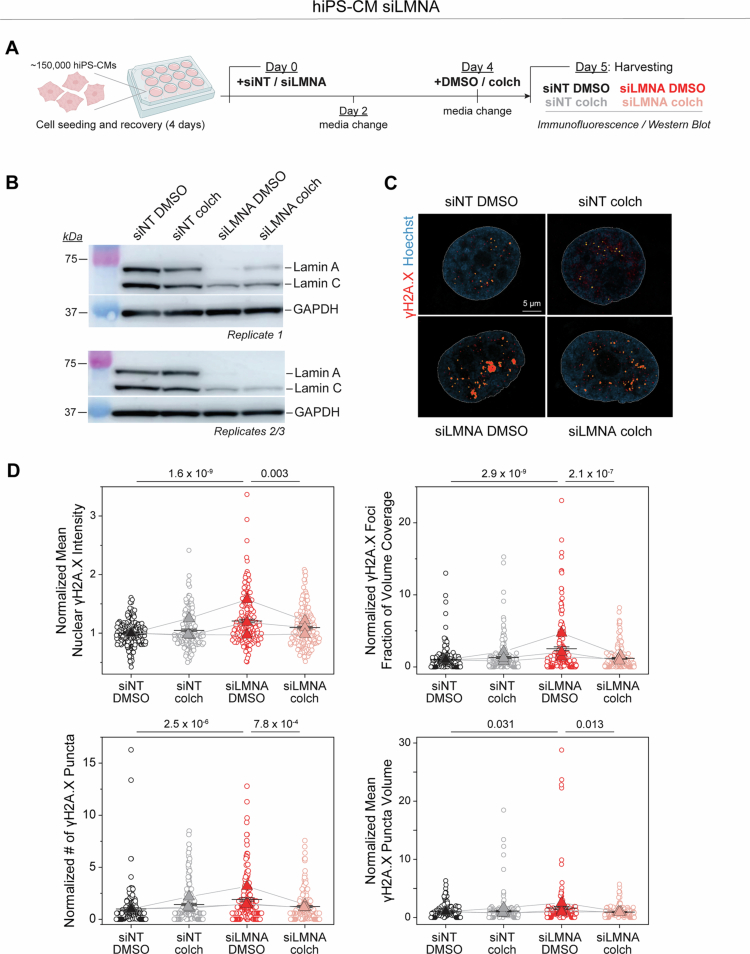
MT disruption protects from DNA damage in LMNA-deficient hiPSC-derived cardiomyocytes. (**a**) Experimental design for the examination of DNA damage in hiPSC-derived cardiomyocytes upon transfection with non-targeted siRNA (siNT) or LMNA-targeted siRNA (siLMNA), and subsequent DMSO or colchicine (colch) treatment. Created in BioRender. Suay Corredera, C. (2025) https://BioRender.com/z6iym3d (**b**) Western blots, separate for replicate 1 and pooled lysates from replicates 2/3 showing the siRNA-mediated lamin A/C knock-down in hiPSC-derived cardiomyocytes. GAPDH was used as a loading control. (**c**) Representative mid-plane images of nuclei (Hoechst) and γH2A.X foci, from the four experimental groups of hiPSC-derived cardiomyocytes. Hoechst was used to outline the nuclei (white line), and the detected γH2AX foci are highlighted in yellow. (**d**) Quantification of mean nuclear γH2A.X intensity and γH2A.X foci fraction of nuclear volume coverage (top). Quantification of the normalized number and volume of γH2A.X foci (bottom). Each replicate is normalized to the mean value of the NT DMSO group. Individual nuclei presented as open circles with mean ± SE of the pooled nuclei. Closed triangles depict replicate means connected by lines to their respective experimental conditions. *N* = 3, *n* = 199 (NT DMSO), *N* = 3, *n* = 197 (NT colch), *N* = 3, *n* = 192 (siLMNA DMSO), *N* = 3, *n* = 203 (siLMNA colch). Statistical significance determined by one-way ANOVA with Bonferroni correction. Source data

We further probed the role of MT forces in a complementary model of *LMNA*-deficient, human induced pluripotent stem cell-derived cardiomyocytes (hiPSC-CMs), which exhibit mechanically induced DNA damage^10,12,13,23^. We transfected hiPSC-CMs with *LMNA*-targeted small interfering RNA (siLMNA) or non-targeted siRNA (siNT) for 5 days and disrupted MTs with colchicine on the last day before cell harvesting (see Experimental Design in Extended Data Fig. 7a). Lamin A/C depletion was confirmed by western blot analysis (Extended Data Fig. 7b), and DNA damage was assessed by immunofluorescence for γH2A.X, an early marker for double-stranded DNA breaks^23^. DNA damage in the *LMNA*-depleted nuclei was significantly reduced upon MT disruption (Extended Data Fig. 7c,d). Taken together, our findings demonstrate that reducing MT forces in lamin A/C-deficient cardiomyocytes protects from nuclear damage.

### MT disruption prevents nuclear damage and preserves cardiac function in mice with cardiac-specific *Lmna* depletion

**Fig. 7:**
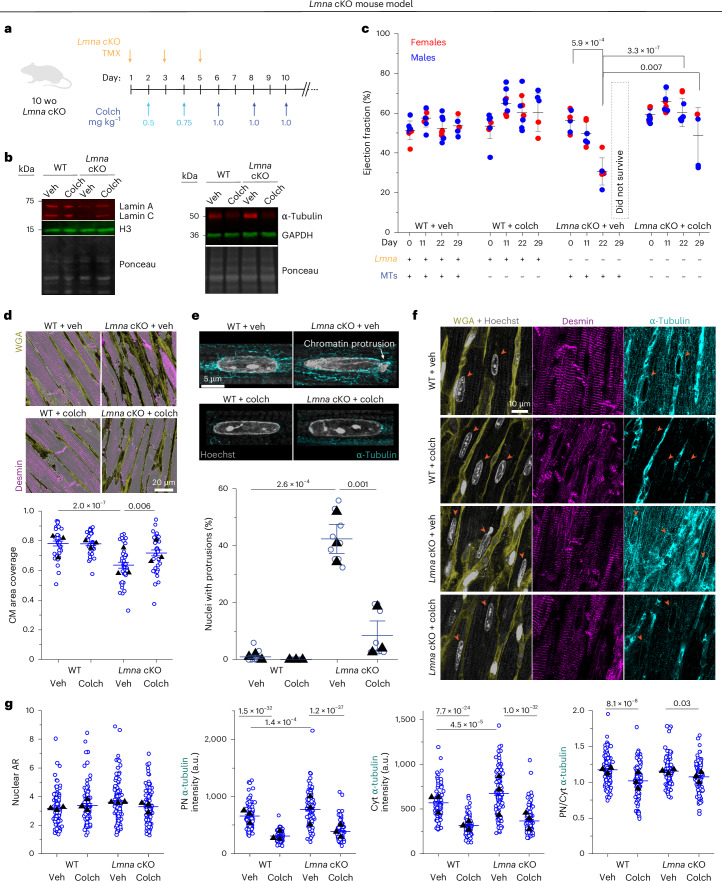
MT disruption preserves cardiac function and protects from nuclear damage in cardiac-specific *Lmna*-depleted mice. **a**, Experimental scheme for concurrent MT depolymerization and cardiomyocyte-specific deletion of *Lmna*. **b**, Representative western blot measuring lamin A/C (left) and α-tubulin (right) protein levels 22 days after first tamoxifen (TMX) injection. **c**, Sequential % ejection fraction measured by echocardiography. Data are shown for pre-injection (day 0) and 11, 22 and 29 days after initial injection. Each data point represents one mouse. Red circles denote females; blue circles denote males. WT + veh: *N* = 8 (d29 *N* = 5), WT + colch: *N* = 9 (d29 *N* = 6), *Lmna* cKO + veh: *N* = 6 (*Lmna* cKO + vehicle-treated mice do not survive to day 29 after injection), *Lmna* cKO + colch: *N* = 9 (d29 *N* = 5). Error bars represent mean ± 1 s.d. Statistical significance was determined by two-way ANOVA with Tukeyʼs multiple correction. **d**, Representative cardiac tissue sections stained for WGA (yellow) and desmin (magenta). Images are MIPs of 4-µm-thick optical sections. Quantification of cardiomyocyte coverage area, inversely related to fibrosis, from WGA channel. Detected cardiomyocyte areas are highlighted on the images with semi-transparent white. Individual blue circles represent individual images; black triangles represent replicate means. *N* = 3 mice per group. WT veh: *n* = 35, WT colch: *n* = 37, *Lmna* cKO + veh: *n* = 39, *Lmna* cKO + colch: *n* = 38 images. Error bars represent mean ± 1 s.e. Statistical significance was determined by one-way ANOVA with Bonferroni multiple correction. **e**, Representative images of cardiomyocyte nuclei (Hoechst, gray) and surrounding MT network (α-tubulin, cyan). Images are MIPs of 4-µm-thick optical sections. Quantification of the percentage of nuclei with chromatin protrusions in each group by three independent, blinded scorers. Open circles represent the percentage of nuclei with chromatin protrusions quantified by each independent user for each animal; closed circles correspond to the mean for each animal. Mean ± s.e. is shown for each group. *N* = 3 mice per group. WT + veh: *n* = 114. WT + colch: *n* = 102. *Lmna* cKO + veh: *n* = 112. *Lmna* cKO + colch: *n* = 113 nuclei. Statistical significance was determined by one-way ANOVA with Bonferroni correction. **f**, Representative cardiac tissue sections stained for WGA (yellow), nuclei (Hoechst, gray), desmin (magenta) and α-tubulin (cyan). Images are MIPs of 4-µm optical sections. Cardiomyocyte nuclei are indicated with arrows. **g**, Quantification of cardiomyocyte nuclei AR, PN, Cyt and PN/Cyt. α-Tubulin intensity. *N* = 3 mice per group. WT veh: *n* = 113. WT colch: *n* = 112. *Lmna* cKO + veh: *n* = 111. *Lmna* cKO + colch: *n* = 112 nuclei. Statistical significance was determined by one-way ANOVA with Bonferroni correction. CM, cardiomyocyte; d, day; wo, weeks old. Panel **a** created with BioRender.com. Source data

**Extended Data Fig. 8:**
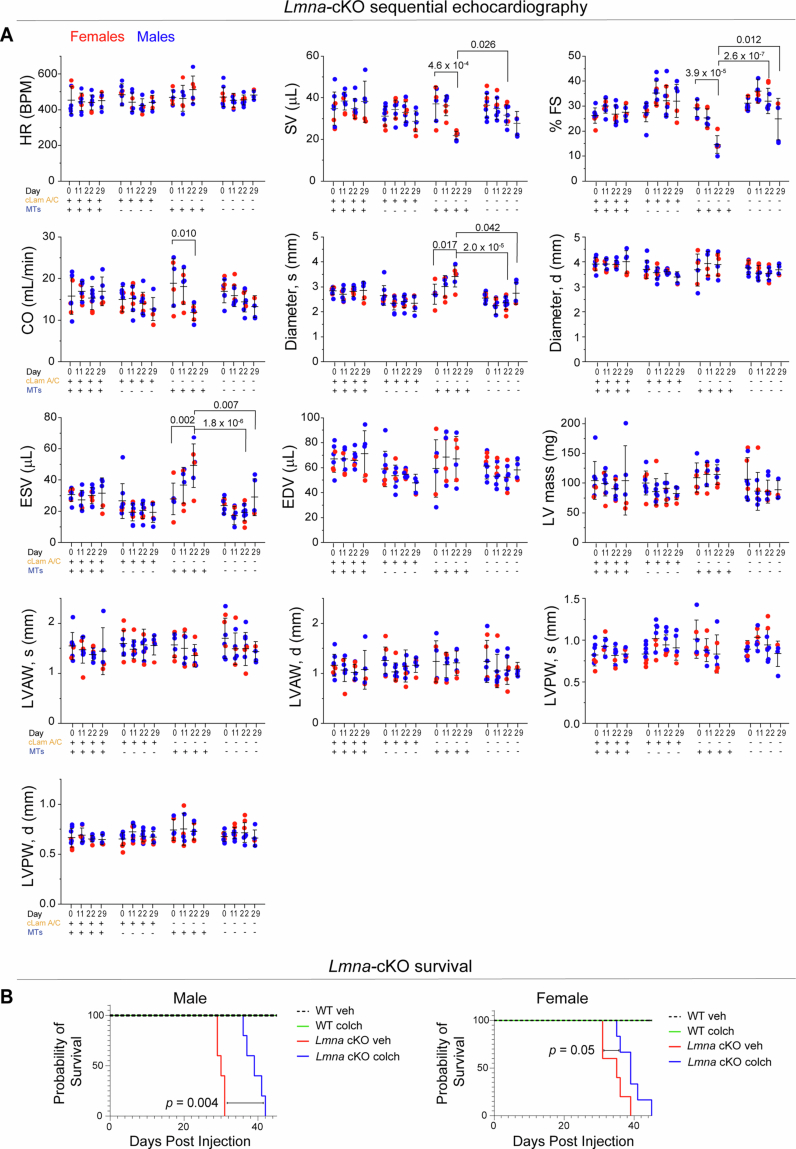
Sequential echocardiography and survival in Lmna cKO mice. (**a**) Sequential echocardiography measurements in cardiac specific *Lmna* depleted mice with in-vivo microtubule disruption. Data shown for pre-injection (day 0), 11, 22, and 29 days post initial injection. Herat rate (HR), stroke volume (SV), % fractional shortening (FS), cardiac output (CO), end-systolic and end-diastolic left ventricular diameters (Diameter, s and d), end-systolic and end-diastolic volume (ESV, EDV), corrected left-ventricular mass (LV mass), end-systolic and end-diastolic left-ventricle anterior and posterior wall thickness (LVAW, s and d, LVPW s and d). Red circles denote females, blue circles denote males. WT + veh: *N* = 8 (day 29: *N* = 5), WT + colch: *N* = 9 (day 29: *N* = 6), *Lmna* cKO + veh: *N* = 6 (*Lmna* cKO + vehicle treated mice do not survive to day 29 post injection), *Lmna* cKO + colch: *N* = 9 (day 29: *N* = 5). Error bars represent mean ± 1 SD. (**b**) Kaplan-Meier survival plots of the different experimental groups. Male: WT+veh: *N* = 2, WT+colch: *N* = 2, *Lmna* cKO+veh: *N* = 5, *Lmna* cKO+colch: *N* = 5. Female: WT+veh: *N* = 3, WT+colch: *N* = 2, *Lmna* cKO+veh: *N* = 5, *Lmna* cKO+colch: *N* = 6. Statistical significance determined by two-way ANOVA with Tukey multiple correction.

To test whether MT disruption can protect from nuclear damage and preserve cardiac function in vivo, we used a mouse model with inducible, cardiomyocyte-specific depletion of lamin A/C (*Lmna* cKO) that we recently generated^24^. Mice at 10 weeks of age were injected with tamoxifen to induce cardiomyocyte-specific *Lmna* depletion^24^ and concomitantly injected with increasing doses of colchicine to disrupt MTs^25^ (Fig. 7a; lamin A/C depletion and MT disruption confirmed in Fig. 7b). We performed serial echocardiography analysis at day 0 (before injections) and at days 11, 22 and 29 after injection. At day 22, we observed significantly reduced cardiac function (left ventricular ejection fraction) in *Lmna* cKO mice (Fig. 7c) as well as indicators of pathological remodeling (left ventricular dilation and hypertrophy; Extended Data Fig. 8a). Notably, *Lmna* cKO mice treated with colchicine had fully preserved left ventricular ejection fraction at 22 days (Fig. 7c) and did not show any evidence of chamber dilation or hypertrophy (Extended Data Fig. 8a). Colchicine-treated mice had improved survival; some colchicine-treated mice exhibited reduced cardiac function only at a later point, 29 days after injection, whereas vehicle-treated *Lmna* cKO mice did not survive until that time (Extended Data Fig. 8b).

**Extended Data Fig. 9:**
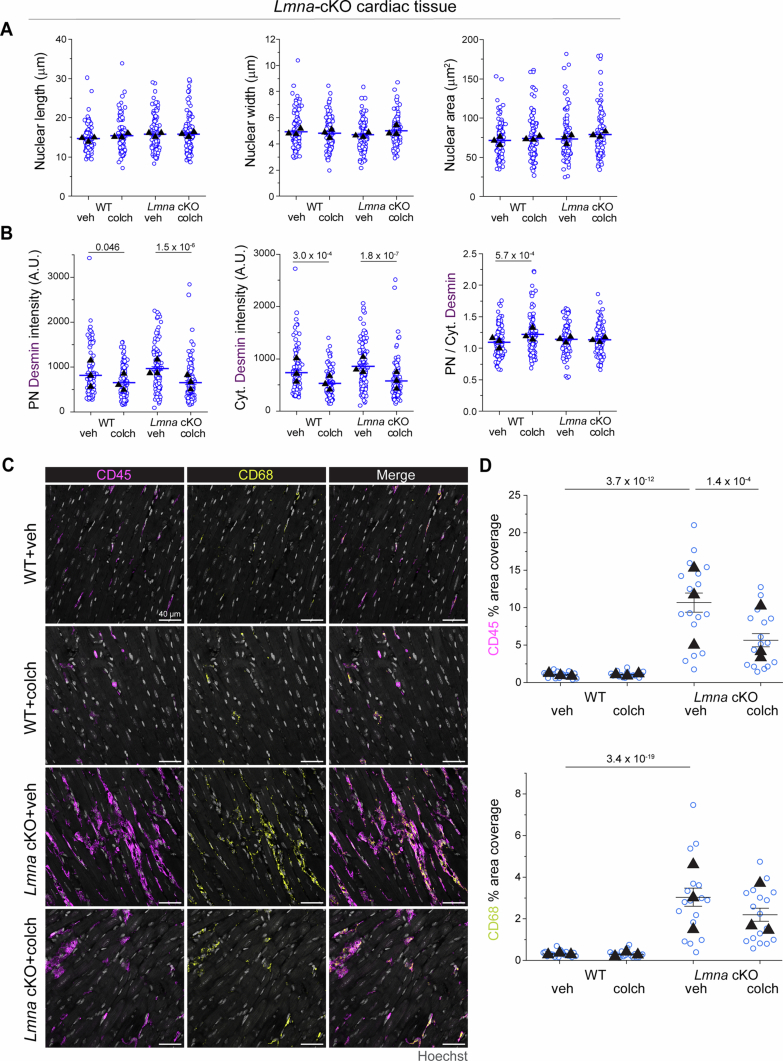
Cellular and immune marker quantification in Lmna cKO mice. (**a**) Quantification of cardiomyocyte nuclear length, width, and area. (**b**) Quantification of perinuclear (PN), cytoplasmic (Cyt) and PN/Cyt. desmin intensity from maximum-intensity-projection images of cardiac tissue sections for the indicated groups. *N* = 3 mice per group. WT veh: *n* = 113. WT colch: *n* = 112. *Lmna* cKO + veh: *n* = 111. *Lmna* cKO + colch: *n* = 112 nuclei. Statistical significance determined by 1-way ANOVA with Bonferroni correction. (**c**) Representative cardiac tissue sections of the indicated groups, stained for CD45 (magenta), a common leukocyte marker, CD68 (yellow), which labels monocytes/macrophages and some other immune cells, and nuclei (Hoechst, gray). Images are maximum intensity projections of 3 μm optical sections. (**d**) Quantification of the percentage coverage area for CD45 and CD68. Blue open circles represent images/myocardial areas. Black triangles represent replicate means. *N* = 3 mice per group. WT + veh: *n* = 18, WT + colch: *n* = 18, *Lmna* cKO + veh: *n* = 18, *Lmna* cKO + colch: *n* = 17 images. Error bars represent mean ± SE. Statistical significance determined by one-way ANOVA with Bonferroni multiple correction.

We analyzed cardiac tissue sections at day 29 after injection (for all groups except *Lmna* cKO mice treated with vehicle control, which were analyzed at day 22). We observed decreased cardiomyocyte area coverage, indicative of increased fibrosis, in *Lmna* cKO mice, in agreement with previous reports^14,24,26^. Colchicine treatment partially restored cardiomyocyte coverage (Fig. 7d). Similar to the *Lmna* N195K model and a previous report^14^, we observed chromatin protrusions specifically at the tips of the *Lmna*-depleted cardiomyocyte nuclei, where the perinuclear MT population is enriched (Fig. 7e, top). Consistent with a rapid and severe *Lmna-*DCM model, we observed approximately 40% of nuclei with chromatin protrusions at day 22 after injection. Notably, in vivo MT disruption robustly protected cardiomyocyte nuclei from chromatin protrusions and damage at day 29 after injection (Fig. 7e), suggestive of potentially even greater effect at comparable day 22. We further characterized nuclear morphology and cytoskeletal organization from cardiac tissue sections and observed no significant changes in nuclear morphology in *Lmna* cKO or colchicine-treated mice (Fig. 7f,g and Extended Data Fig. 9a). As expected, in vivo colchicine injections significantly reduced the perinuclear and cytoplasmic MT populations and specifically reduced enrichment of the perinuclear MT cage (Fig. 7f,g). Colchicine treatment also reduced the density of desmin intermediate filaments in both WT and *Lmna* cKO mice (Extended Data Fig. 9b). Markers of immune cell activation were increased in the *Lmna* cKO cardiac tissue, consistent with previous reports^14^, and were reduced upon colchicine treatment (Extended Data Fig. 9c,d). This effect could be due to the reduction in nuclear ruptures and downstream activation of inflammatory signals or direct anti-inflammatory properties of colchicine.

Collectively, these findings are consistent with our observations from the *Lmna* N195K mutant model and demonstrate that disruption of the perinuclear MT cage protects from nuclear damage and is sufficient to preserve cardiac function in *Lmna* cKO mice.

### Computational model of nuclear damage due to resting cytoskeletal forces

**Fig. 8:**
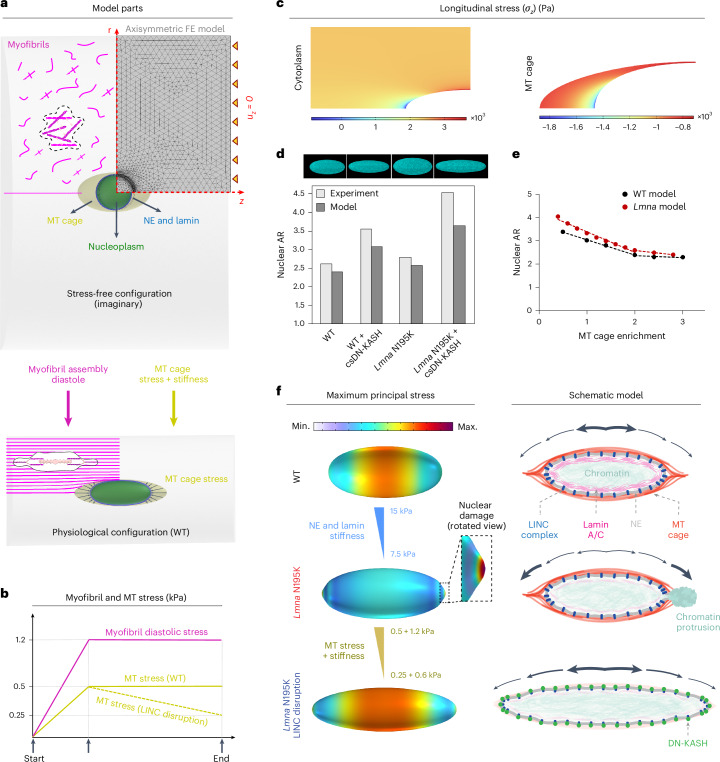
A computational model to explain nuclear damage in resting *LMNA* mutated cardiomyocytes and its rescue through LINC complex disruption. **a**, Axisymmetric finite element (FE) model considering an imaginary stress-free configuration (top) for cardiomyocytes consisting of a round nucleus (which is further divided into nucleoplasm and NE and its underlying lamina), an ellipsoid MT cage and the surrounding randomly distributed unassembled myofibrils. Diastolic contractility, geometric constraints of the myocardium, restoring forces of titin proteins and compressive MT forces work in concert to deform this initial configuration to the physiological stressed configuration (bottom) where the nucleus is elongated and the myofibrils are aligned. **b**, Variations of the myofibril diastolic stress and MT compressive stress over the simulation time. **c**, Distribution of the longitudinal stress component in the cytoplasm and the MT cage in physiological conditions. **d**, Images of simulated nuclei (top) and comparison between the model predictions and the experimental data for nuclear aspect ratio for the four experimental groups (bottom). **e**, Simulation results for variation of the nuclear aspect ratio as a function of MT enrichment for WT and *LMNA* mutated (10 kPa) groups. Closed circles represent simulation data points; dashed lines represent piecewise linear regression fits for each group. **f**, Simulations show that, beyond a critical value for the MT compressive stresses, a form of instability emerges at the nuclear tips in which the location of maximum principal stress shifts from the middle of the nucleus to the tips (left, top and middle). Continuing the simulation after the instability by reducing MT compressive forces (simulating LINC complex disruption) shows that the nucleus becomes thinner and longer, with the maximum principal stress returning to the middle part (left, bottom). These observations are schematically shown in the right panel.

**Extended Data Fig. 10:**
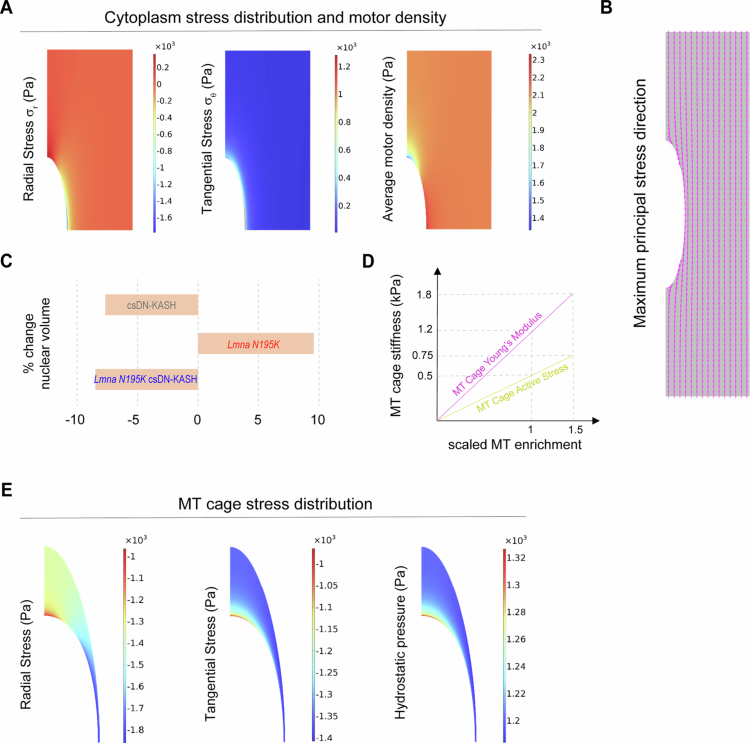
Simulation parameters and results for the computational model describing perinuclear stress distribution in mature cardiomyocytes. (**a**) Simulation results depicting stress distribution and average myosin motor density in the cardiomyocyte cytoplasm under physiological conditions. Radial stress component, tangential stress component and average motor density defined as $({\rho }_{11}+{\rho }_{22}+{\rho }_{33})/3$. (**b**) Model predictions for the maximum principal (maximum tensile) stress direction in the cytoplasm. (**c**) In vivo % volume changes relative to WT conditions. (**d**) Variations of the MT cage stiffness and active stress to simulate MT enrichment around the nucleus. (**e**) Simulation results for stress distribution in the microtubule cage in WT conditions. Radial stress component (Pa), Tangential stress component (Pa) and hydrostatic pressure (Pa).

To better understand the forces that dictate nuclear morphology and damage, we developed a computational finite element axisymmetric model for the resting, mature cardiomyocyte (Fig. 8a). In this model, we explicitly consider the nucleus (subdivided into nucleoplasm and NE with lamina), its surrounding MT cage and the myofibrils (cytoplasm) as components involved in nuclear deformations. We first consider a cylindrical stress-free (imaginary) cardiomyocyte configuration with round nucleus, misaligned myofibrils and restricted axial displacement (*u*_*z*_ = 0) of the cell ends (Fig. 8a and Extended Data Fig. 10; see Methods for more details). We add an isotropic and homogenous compressive stress field (*σ*_MT_) to the perinuclear MT cage^3^ and assume that, in the stress-free configuration, the contractility of the randomly distributed actomyosin fibers (*ρ*_*ij*_, where *i* and *j* denote the cartesian components of a vector) is initially isotropic and uniform everywhere in the cytoplasm (*ρ*_0_). Note that *ρ*_*ij*_ shows the cardiomyocyte contractility at rest (diastole)^27^, which differs from its active contractility during systole.

To induce the cytoskeletal pre-stress field of mature, rod-shaped cardiomyocytes^3,28,29^, we increase *ρ*_0_ and *σ*_MT_ from 0 to reach the physiological stressed (WT) configuration (Fig. 8a,b). As a result, the cytoplasm contracts in the radial direction while its length remains constant (Supplementary Video 3), leading to the development of an anisotropic stress field within the cell, whose direction is perfectly aligned with the direction of aligned myofibrils in the mature cardiomyocyte (Fig. 8a, bottom, and Extended Data Fig. 10b). Our simulations show that myofibril maturation imposes substantial lateral compressive forces on the nucleus, leading to nuclear elongation in the axial direction (Fig. 8a, bottom). Nuclear elongation is resisted by elastic compression of surrounding MTs and their pushing forces (*σ*_MT_). Together, these interactions induce the pre-stress field in quiescent cardiomyocytes (Extended Data Fig. 10a), even in the absence of cell contractility, although cell contractility might further increase the stress on the nucleus. Our model predicts that maximum and minimum myofibril tensions happen at the short and long tips of the MT cage, respectively, whereas, far from this cage, tension is nearly constant (Fig. 8c, left). By contrast, maximum and minimum axial MT compression happens at the long and short tips of the nucleus, respectively (Fig. 8c, right). The obtained values for diastolic tension of myofibrils (2–3 kPa) and axial compression of MTs (1–1.8 kPa) are in good agreement with their corresponding experimental estimations^27–29^.

Figure 8f depicts model predictions for nuclear morphology changes due to *LMNA* mutations (reduced stiffness of the NE and lamina^30,31^) and LINC complex disruption (decreased stress and stiffness of the MT cage, based on our experimental observations). Our simulations reveal that LINC complex disruption increases the nuclear aspect ratio, which is further exacerbated upon *LMNA* depletion, closely matching our experimental findings and reinforcing the role of MTs in shaping nuclear morphology. By changing the cage stress and stiffness (Extended Data Fig. 10d and Methods), our simulations reproduced the experimentally observed biphasic relationship between nuclear aspect ratio and perinuclear MT enrichment (Fig. 8e).

Furthermore, for any specified stiffness of the NE and lamina, a critical value for MT enrichment exists, beyond which a form of instability emerges at the nuclear tips (Fig. 8f). Interestingly, the instability appears concomitantly with a shift in the maximum principal stress (tension) from the middle of the nucleus to its long tips (Fig. 8f). The combination of diluted lamina at high-curvature nuclear tips^32^ with high tension at these locations increases the probability for nuclear lamina gaps to form, ultimately facilitating nuclear damage and rupture^33^. Together, these simulation results suggest that compressive forces of the MT cage are sufficient to drive nuclear damage in mature, quiescent *LMNA*-deficient cardiomyocytes.

After the appearance of instability at the nuclear tips, we simulated LINC complex disruption by decreasing stress and stiffness of the MT cage (Fig. 8b). As a result, the instability disappears, and the nucleus becomes thinner and longer while the location of the maximum principal stress comes back to the middle of the nucleus (Fig. 8f, bottom). This provides a quantitative explanation for rescue of nuclear damage by LINC complex disruption in *LMNA*-deficient cardiomyocytes.

Overall, our experimental and modeling data suggest that nuclear elongation is regulated by the balance between cross-sectional myofibril compression and the perinuclear MT cage that provides axial compressive forces to resist elongation (Fig. 8f, right). A compromised lamina leads to redistribution of the maximum principal stress to nuclear tips, which contributes to NE rupture and chromatin protrusions. Decoupling the nucleus from MT-based forces via LINC complex disruption or direct MT disruption redistributes the stress away from the tips, thus protecting the nucleus from new ruptures.

## Discussion

This study introduces a method to quantify sarcomere–nuclear strain coupling in beating cardiomyocytes and investigates its role in *Lmna* cardiomyopathy. We found no evidence to support active sarcomere contraction as a primary cause of nuclear damage in lamin-deficient cardiomyocytes. Instead, damage was linked to forces generated by the dense cage of perinuclear MTs and kinesin motors. Either LINC complex or MT disruption protected from NE ruptures and DNA damage, and in vivo MT disruption preserved nuclear integrity and cardiac function and extended survival in mice upon cardiac-specific lamin A/C depletion. Computational modeling further demonstrated that reducing MT forces redistributed mechanical stress away from local vulnerabilities at the tips of lamin-deficient nuclei, implicating MT–nuclear interactions as a therapeutic target in laminopathy.

To our knowledge, our active strain coupling assay is the first to capture physiologically relevant nuclear strains in mature, electrically stimulated cardiomyocytes. Previous studies in passive stretch models^1,34^ or immature cardiomyocytes^28,35^ provided inconsistent insights on cytoskeletal–nucleoskeletal coupling. We observed more than 10% sarcomere strain with tightly coupled, although dampened, nuclear deformation. Surprisingly, LINC complex or MT disruption had only mild effects on strain coupling. This suggests that nuclear deformation is primarily driven by surrounding myofibrils that squeeze the nuclei during contraction, largely independent of direct physical connection via LINC complexes.

We used both acute in vitro (AdV DN-KASH) and chronic in vivo (csDN-KASH) LINC disruption. Although each disrupted the MT cage and led to nuclear elongation, they did so to different degrees, and AdV DN-KASH reduced strain coupling while also expanding nuclei along their short axis. This may be due to differential cytoskeletal and nuclear remodeling in response to rapid in vitro versus chronic in vivo LINC complex disruption. For example, chronic csDN-KASH led to more complete reduction of the MT cage and greater nuclear elongation, consistent with the relationship between MT cage enrichment and nuclear aspect ratio. The lack of expansion in the nuclear short axis upon in vivo csDN-KASH could arise from greater nuclear confinement from myofilaments in vivo, among other factors.

Although *Lmna* N195K cardiomyocytes exhibited an approximately 30% increase in integrated nuclear strain during the contractile cycle (Fig. 4h), this did not readily explain nuclear damage or functional decline. LINC complex disruption preserved cardiac function and nuclear integrity without normalizing nuclear strain, and increased contractile load did not increase nuclear ruptures in lamin-deficient cardiomyocytes. However, this latter result is limited by short stimulation times ex vivo, and chronic in vivo studies with augmented workload are required to further assess contractile involvement in NE ruptures.

Our experimental and modeling results converge on MT forces regulating nuclear morphology in adult cardiomyocytes. During maturation, cardiomyocytes undergo uniaxial elongation, and centrally located nuclei are squeezed by aligning myofibrils, causing nuclear elongation^29^. The perinuclear MT cage provides balancing compressive forces that resist further elongation. Reducing MT cage density below a threshold (Fig. 5c) allows elongation, particularly in more deformable lamin-deficient nuclei. However, elongation per se does not appear pathological; *Lmna* N195K csDN-KASH mice have dramatically elongated yet intact nuclei and preserved cardiac function.

Nuclear elongation was not significantly different between WT and *Lmna* N195K cardiomyocytes with intact MT cages, differing from previous reports in other laminopathy models^7,8^. This may reflect differences in cytoskeletal remodeling or disease severity or imaging resolution and selection criteria. With high-resolution imaging, we excluded nuclei with chromatin protrusions from shape analysis (20% in *Lmna* N195K group). With lower-resolution imaging and no exclusion, we indeed observed mild (11%) but statistically significant elongation in *Lmna* N195K nuclei (Extended Data Fig. 2b). Regardless, our observation of nuclear ruptures without significant elongation aligns with recent reports from *Lmna*-depleted cardiomyocytes^14,26^.

LINC complex disruption reduced perinuclear MTs and kinesin-1 motors concomitant with reduced NE damage. Kinesin-1 depletion is protective in *Lmna-*deficient developing skeletal muscle myotubes, where nuclear damage is driven by MT forces associated with kinesin-1-dependent nuclear movements^10^. Cardiomyocyte nuclei are largely static, and damage appears to be driven by compressive forces from the MT cage. Despite this mechanistic distinction, convergent strategies that disrupt kinesin–nuclear interactions warrant examination broadly for striated muscle laminopathies.

We used a rapid *Lmna* depletion mouse model to test the potential of MT disruption in preserving nuclear integrity and cardiac function. We identified chromatin protrusions in approximately 40% of *Lmna*-depleted cardiomyocyte nuclei, consistent with recent studies^14,26^, and localized chromatin protrusions to the tips of nuclei encaged by perinuclear MTs. Colchicine reduced perinuclear MTs, protected nuclei from damage, improved cardiac function and remodeling and extended lifespan. Although these results support targeting perinuclear MTs to prevent *LMNA* cardiomyopathy progression, we have not tested the ability of MT disruption to rescue cardiac function in already developed cardiomyopathy. Furthermore, colchicine’s dose-limiting toxicity and high murine dosage limit translational relevance. Safer, more precise strategies targeting MT–nuclear interactions are needed.

Many experimental models are available to study cardiac laminopathies^36,37^, and we used several herein. Although human iPSC-CM models are a powerful tool and exhibit *LMNA*-associated DNA damage, they have rounder nuclei and lack nuclear ruptures, likely due to an immature cytoskeleton and nucleoskeleton, limiting the utility of this model^12,38,39^. Among mouse models, *Lmna* depletion represents the most rapidly progressing DCM model, whereas *Lmna* N195K and *Lmna* H222P variants exhibit slower progression and differing severity; yet ultimately all lead to progressive DCM with contractile disfunction and fibrosis. Nuclear abnormalities are a common theme across models but vary in presentation^8,40–42^. Although in vivo disruption of MTs protected from the severe nuclear damage with lamin A/C depletion, nuclear stability is only mildly impaired in *Lmna* H222P myocytes. It remains to be determined whether LINC complex or MT disruption will improve cardiac function in this model.

*Lmna* N195K females survived longer than their male counterparts (approximately 20%), aligning with sex differences observed in patients. Females exhibit fewer malignant ventricular arrhythmias and instances of end-stage heart failure, leading to less overall mortality^43^. In mouse models of laminopathy, this has been attributed to nuclear accumulation of androgen receptor and its cofactors^44^. Future research may provide insight into sex-specific disease drivers and therapeutic possibilities.

We focused on mechanical contributors to nuclear damage and *LMNA* pathogenesis. Lamina roles in chromatin organization and gene expression likely also contribute to pathology and may exhibit interdependence with altered nuclear mechanical stability^45,46^.

*Lmna* N195K csDN-KASH mice demonstrated preserved cardiac function at 10 weeks and 12 weeks of age but still succumbed to early mortality at approximately 17 weeks, in alignment with a previous report of increased lifespan by 5–6 weeks after SUN1 deletion in laminopathy mice^15^. Although the ultimate cause of death is undetermined, this may involve other cell types contributing to pathophysiological cardiac remodeling, conduction issues, a loss in myocyte-to-non-myocyte coupling or non-cardiac comorbidities, such as skeletal muscle defects. We cannot rule out or attribute causation to any of these factors. For AdV DN-KASH effects, we cannot rule out other potential functions of KASH overexpression at the NE.

We observed extended survival for *Lmna* cKO mice treated with colchicine; however, colchicine treatment by itself is toxic and contributes to mortality over time, precluding extended survivability assessments. These studies thus serve as proof of concept for targeting MT–nuclear interactions, and, for clinical applications, more refined treatment approaches are likely needed.

Overall, this study introduces a method to investigate sarcomere–nuclear strain coupling in the beating cardiomyocyte and implicates MT forces in *LMNA*-linked nuclear damage. Targeting specific interactions between MTs and the LINC complex should be pursued as a potential cardioprotective strategy in *LMNA* cardiomyopathy.

## Methods

### Animals

Animal care and use procedures were performed in accordance with the standards set forth by the University of Pennsylvania Institutional Animal Care and Use Committee and the Guide for the Care and Use of Laboratory Animals published by the US National Institutes of Health. Protocols were approved by the University of Pennsylvania Institutional Animal Care and Use Committee. All animals provided by the Lammerding laboratory at Cornell University were bred and maintained according to relevant guidelines and ethical regulations approved by the Cornell University Institutional Animal Care and Use Committee (protocol 2011-0099). Both rats and mice were housed in a facility with 12-hour light/dark cycles and provided ad libitum access to water and chow. Temperature and humidity were checked daily to ensure that these parameters stayed within appropriate ranges (20–26 °C and 30–70%, respectively).

In csDN-KASH mice, a dominant negative KASH domain of nesprin-2 tagged with green fluorescent protein (GFP) is expressed in cardiomyocytes upon tamoxifen treatment^18^. We confirmed successful csDN-KASH induction after tamoxifen treatment by scoring the appearance of a csDN-KASH–GFP ring that surrounded the nucleus in more than 95% of cardiomyocytes, consistent with previous estimates of induction efficiency with this model^18^. *Lmna*^N195K/N195K^ (ref. ^9^) and csDN-KASH mice were backcrossed at least seven generations into a C57BL/6 line^10^. To generate αMHC Cre^+/−^KASH^+/−^*Lmna*^N195K/N195K^ mice (*Lmna* N195K csDN-KASH), male αMHC Cre^+/−^*Lmna*^N195K/+^ and female KASH^+/−^*Lmna*^N195K/+^ mice were crossed to create experimental and control littermate mice. All mice were maintained in a uniform C57BL/6 background. *Lmna* mutant mice were provided with gel diet supplement (BioServe, Nutri-Gel Diet) to improve hydration and overall health and quality of life. To induce cardiomyocyte-specific KASH-mediated LINC complex disruption, 30 mg kg^−1^ tamoxifen (Cayman Chemical, 13258) suspended in sunflower oil (Sigma-Aldrich, 88921) was injected intraperitoneally daily for five consecutive days, starting at approximately 3 weeks of age, followed by a 1-week washout. Controls included αMHC Cre^+/−^KASH^+/−^*Lmna*^N195K/N195K^ mice treated with vehicle and *α*MHC Cre^+/−^KASH^+/−^*Lmna*^+/+^ mice littermates.

To generate *Lmna*^N195K/N195K^ mice expressing cGAS–tdTomato, the previously described cGAS/MB21D1–tdTom transgenic mouse^10^ was crossed into the *Lmna* N195K background to generate 3×Flag-cGAS^E225A/D227A^–tdTomato-positive *Lmna*^N195K/N195K^ mice within two generations. All mice were maintained in a uniform C57BL/6 background. Single cardiomyocyte data were collected at stated timepoints. Controls included *Lmna*^+/+^ mice expressing the cGAS construct.

To generate the inducible, cardiac-specific *Lmna* deletion mouse model (*Lmna* cKO), αMHC-MerCreMer (The Jackson Laboratory (JAX), strain number 005657) and *Lmna* floxed mice (JAX, strain number 026284) were crossed, as previously described^24^, to obtain αMHC Cre^+/−^*Lmna*^fl/fl^ mice. αMHC Cre^+/−^*Lmna*^+/+^ littermates served as controls (*Lmna* cWT). All mice were maintained in a uniform C57BL/6 background. To induce lamin A/C depletion in cardiomyocytes, tamoxifen was dissolved in sunflower oil (Sigma-Aldrich, 88921) to a concentration of 30 mg kg^−1^ and injected intraperitoneally at 10 weeks of age every other day for three total injections (days 1, 3 and 5). Vehicle-only injections served as additional controls. To disrupt MT in vivo, mice were also injected in between tamoxifen doses with increasing doses of colchicine (days 2, 4, 6, 8 and 10 euthanization; see concentration in Fig. 6f). PBS of equal volume was intraperitoneally injected for controls. Lamin A/C depletion was validated by western blot on cardiac tissue obtained from mice at 22 days after initial tamoxifen injection, using anti-lamin A/C (Santa Cruz Biotechnology, sc-376248; 1:1,000) and anti-H3 (Cell Signaling Technology (CST), 44995; 1:5,000) as loading control. MT disruption in the heart was validated by western blot using anti-α-tubulin (Abcam, ab7291; 1:3;000) and GAPDH (CST, 2118S; 1:5,000) as loading control. At this timepoint, lamin A/C levels were reduced approximately 58%, similar to recent reports^14,24,26^ and consistent with the long half-life of lamin proteins. This depletion mimics the reduced lamin A/C levels seen in multiple human *LMNA* cardiomyopathy models^47^.

### Echocardiography

Mice were anesthetized with 2% isoflurane and placed on a stereotactic heated scanning base (37 °C) attached to an electrocardiographic monitor. Left ventricular structure and function were determined with a Vevo 2100 imaging system (VisualSonics) equipped with a MS550D transducer (22–55 MHz). Echocardiographic parameters were measured for at least five cardiac cycles using AM mode images. Analysis was performed using AutoLV Analysis software (VisualSonics) and conducted by observers blinded to the mouse genotype and/or treatment groups.

### Adult rat and mouse cardiomyocyte isolation and culture

Primary adult ventricular myocytes were isolated from 8–12-week-old Sprague Dawley rats or 8–9 week-old mice using Langendorff retrograde aortic perfusion with an enzymatic solution as previously described^20^. In brief, the heart was removed from an anesthetized rodent under isoflurane and retrograde perfused on a Langendorff apparatus with a collagenase solution. The digested heart was then minced and triturated with glass pipettes to free individual cardiomyocytes. The resulting supernatant was separated and centrifuged at 17*g* (300 r.p.m.) to isolate cardiomyocytes. These cardiomyocytes were then resuspended in cardiomyocyte media (Medium 199 (Thermo Fisher Scientific) supplemented with 1× insulin-transferrinselenium-X (Gibco), 1 μg μl^−1^ primocin (InvivoGen) and 20 mM HEPES (pH 7.4)) (UPenn Cell Center) at low density, cultured at 37 °C and 5% CO_2_ with the addition of 25 μmol l^−1^ cytochalasin D in the media.

### Active sarcomere–nuclear strain coupling

Active strain coupling was quantified by back-to-back measurement of sarcomere length contractility and nuclear deformation during electrically stimulated contractions in adult cardiomyocytes (no blebbistatin was added to allow for actin-myosin contractility). Cardiomyocytes in culture media were loaded with 4 µM Hoechst and transferred to a custom-fabricated cell chamber (IonOptix) mounted on a Zeiss LSM 880 inverted confocal microscope with ×63 oil 1.4 numerical aperture objective. Experiments were conducted at room temperature, and field stimulation was provided at 1 Hz with a cell stimulator (IonOptix, MyoPacer). Rod-shaped cells with stable contractions were selected, and baseline sarcomere length >1.7 µm and sarcomere length strain >10% were used as inclusion criteria. For each cell, sarcomere length contractility was first measured with a transmitted light camera (IonOptix, MyoCam-S) and real-time optical Fourier transform analysis (IonOptix, IonWizard). For each cell, five steady-state and consistent sarcomere length traces were recorded in a region of interest (ROI) close to the nucleus, either above or below the nucleus. The microscope was then immediately switched to fast mode Airyscan confocal, and Hoechst fluorescence was imaged with a 405-nm laser (lowest laser power) at 11 milliseconds per frame (91 Hz) for five additional steady-state contractions. Initial image analysis was performed using ZEN Black software for Airyscan processing. Stimulation times were recorded with the sarcomere contractility and nuclear imaging files for offline alignment of the traces. An average sarcomere contractility trace was generated for each cell (IonOptix, IonWizard) and exported to MATLAB (MathWorks, R2022b) for further alignment with nuclear deformation traces. Nuclear image analysis was performed with Arivis V4D 4.0–4.1 by a pipeline to autosegment (Otsu) the nucleus object in each frame and export nuclear morphology parameters. Nuclear length and width traces were imported to MATLAB, averaged for each cell and aligned with the corresponding average sarcomere contractility trace (after interpolation to 91 Hz to match nuclear time trace). Sarcomere and nuclear strains were calculated by dividing the corresponding instantaneous strains by the baseline lengths prior to stimulation. For each experimental group, 15–20 cardiomyocytes were recorded from 3–4 biological replicates. Systolic dampening was calculated as the integrated area under the curve during contraction with respect to the linear relationship (Fig. 1g, left). Diastolic dampening is the integrated area under the curve during re-lengthening with respect to a linear relationship with the intercept fixed at end-systolic strain (Fig. 1g, right).

### AdV DN-KASH

Adult rat cardiomyocytes were transduced for 48 hours with previously validated adenovirus overexpressing a dominant negative KASH peptide that prevents interactions between endogenous nesprin and SUN proteins^1,3^.

### hiPSC-CMs

We purchased hiPSC-CMs from Ncardia (Nc-C-BRCM, lot number 6280620G20). Cells were maintained in RPMI supplemented with 10% FBS (Gibco, 16000044), 1% penicillin–streptomycin (Gibco, 15140122) and 2% B27 (Gibco, 17504044). After 4 days in maintenance media, 150,000 cells were plated onto glass coverslips (for immunofluorescence) or into 12-well culture dishes coated with 10 μg ml^−1^ fibronectin (Sigma-Aldrich, F1141) diluted in DPBS with Mg^2+^ and Ca^2+^. Cells were then allowed to recover for 4 days in maintenance media. On day 0, cells were treated with siRNAs against either a non-targeting control (siNT) or lamin A/C (siLMNA) at a concentration of 5 nM using Lipofectamine RNAiMAX transfection reagent diluted in Opti-MEM. Cells were incubated with siRNAs for 5 days total, and media were changed on days 2 and 4. On day 4, cells were also treated with either DMSO as a control or colchicine at a concentration of 1 µM. Twenty-four hours after DMSO or colchicine treatment, cells were harvested for downstream analysis.

### Immunofluorescence of isolated cardiomyocytes

Primary rat and mouse cardiomyocytes were fixed in 4% paraformaldehyde (PFA) (Electron Microscopy Sciences) for 10 minutes, washed three times with PBS and permeabilized in 0.1% Triton X-100 for 10 minutes at room temperature. After washing twice with PBS, cells were placed in blocking buffer (1:1 Seablock (Abcam) and 0.1% Triton X-100 (Bio-Rad)) in PBS for at least 1 hour at room temperature and then labeled with primary antibodies (see below) for 24–72 hours at 4 °C. Cells were then washed three times in PBS and then labeled with secondary antibodies in PBS at room temperature for 2–4 hours. Hoechst was added for the last 10 minutes, of secondary incubation, and cells were washed twice with PBS. Stained cells were mounted on Number 1.5 coverslips in ProLong Diamond Antifade Mountant (Thermo Fisher Scientific) for imaging. Slides were left to cure in the dark for at least 24 hours prior to imaging. Next, hiPSC-CM coverslips were fixed in 4% PFA for 10 minutes at room temperature and then rinsed in DPBS for 5 minutes three times. Cells were permeabilized with 0.5% Triton X-100 for 10 minutes, blocked in 1% BSA in PBST (8 mM Na_2_HPO_4_, 150 mM NaCl, 2 mM KH_2_PO_4_, 3 mM KCl and 0.05% Tween 20 (pH 7.4)) and incubated with primary and secondary antibodies diluted in PBST + 1% BSA for 1 hour each at room temperature. Samples were counterstained with DAPI solution (Sigma-Aldrich, D9542) for 10 minutes at room temperature and then rinsed with PBS and stored/imaged in SlowFade Gold Antifade Mountant (Invitrogen, S36936).

### Mouse tissue processing and staining

Immediately after tissue collection, hearts were washed with 1× PBS and fixed in 4% PFA (diluted in 1× PBS) at 4 °C overnight. For immunofluorescence analysis, samples were washed with PBS and flash frozen in Tissue-Tek O.C.T. Compound (Sakura, 4583) in an ethanol bath, followed by storage at –70 °C. Frozen tissue blocks were cryosectioned using an Epredia Microm HM525 NX Cryostat (SN: S20020277) to a thickness of 5 μm, mounted on 25 × 75 × 1.0-mm Superfrost Plus microscope slides (Fisherbrand, 12-55015) and left to air dry for 1 hour, followed by blocking and permeabilization with a solution of 3% BSA and 5% filtered horse serum in PBST (0.05% Triton X-100 and 0.03% Tween (Sigma-Aldrich) in 1× PBS) for 1 hour at room temperature.

For Picrosirius red staining, whole ventricles were cryosectioned at 5-μm intervals and left to air dry for at least 1 hour at room temperature and stored at −20 °C. Upon processing, slides were brought to room temperature and put in Bouin’s fixative (Electron Micrscopy Sciences, 15990-10) set in a 56 °C water bath for 15 minutes and then washed two times with Milli-Q water. Slides were then submerged in Sirius red (Sigma-Aldrich, 365548-5G, Direct Red 80 in 1.3% picric acid, and Sigma-Aldrich, P6744-1GA) for 2 hours, gently rocking at room temperature. Slides were washed two times in 0.5% acetic acid and then dehydrated using 95% and then 100% ethanol. Finally, slides were equilibrated in xylene substitute and mounted in xylene-based media (Leica, 3801731) and imaged using ScanScope Cs2 from Aperio and analyzed using the color deconvolution macro in Aperio’s analysis software package.

### Immunofluorescence of cardiac tissue sections

Five-micrometer-thick sections were placed on glass slides and processed for immunofluorescence as described in ref. ^48^, with modifications. The paraffin wax was melted, and deparaffinization was achieved by 2 × 20-minute washes in xylene (StatLab, 8400-1). Samples were rehydrated through sequential 2-minute washes in decreasing concentrations of ethanol (100%, 95%, 75% and 50%), followed by ddH_2_O. Then, glass slides were placed in 1× reveal decloaker solution (Biocare Medical, RV1000M), and heat antigen retrieval was performed for 15 minutes at high pressure using a pressure cooker (Instant Pot Pro 10-in-1 pressure cooker, 8 quart). After cooling, residual reveal decloaker solution was washed off in ddH_2_O, and a hydrophobic ring was hand drawn surrounding the sections using a Super PAP pen (Electron Microscopy Sciences, 71312). Sections were washed in 1× PBS and permeabilized with 1× PBS + 0.25% Triton X-100 for 10 minutes. After permeabilization, sections were thoroughly washed in 1× PBS. An additional wash in 0.1% PBST (0.1% Tween 20 in 1× PBS) preceded blocking for 1 hour at room temperature in blocking solution (3% BSA (Sigma Aldrich, A7906) in 0.1% PBST).

Incubation in primary antibodies was performed in blocking solution for 24 hours (goat polyclonal anti-CD45, R&D Systems, AF114, 1:50, and rabbit monoclonal anti-CD68, CST, 97778, 1:100) or 48 hours (for mouse monoclonal anti-α-tubulin, Sigma-Aldrich, T5168, clone B-5-1-2, 1:50, and goat polyclonal anti-desmin, R&D Systems, AF3844, 1:250), at room temperature, in a homemade humidified chamber. Sections were rinsed in 1× PBS and washed 2 × 15 minutes with 0.1% PBST. Then, incubation in secondary antibodies (donkey anti-goat IgG AF647, Invitrogen, A21447, 1:100; donkey anti-mouse IgG AF568, Invitrogen A10037, 1:100; and donkey anti-mouse IgG AF488, Invitrogen A21206, 1:100) was performed in 0.1% PBST for 24–48 hours, at room temperature, protected from the light in the humidified chamber. AF488-conjugated wheat germ agglutinin (WGA) (Invitrogen, W11261) was added during the secondary antibody incubation (1:500, final concentration 25 μg ml^−1^) when pertinent. The sections were rinsed in 1× PBS and stained for 20 minutes with Hoechst 33342, Trihydrochloride, Trihydrate (Invitrogen, H3570; final concentration 10 μg ml^−1^ in 1× PBS). Sections were thoroughly washed in 1× PBS before mounting in ProLong Diamond Antifade Mountant (Invitrogen, P36961).

### Antibodies, labels and pharmaceuticals

The following reagents were used: Hoechst 33342 (Invitrogen, H3570); SPY650-DNA, SPY555-tubulin and SiR-actin (0.1 µl ml^−1^, Spirochrome); anti-α-tubulin mouse monoclonal antibody, clone DM1A (1:500; Abcam, ab264493); anti-KIF5B rabbit monoclonal antibody clone EPR10276(B) (1:500; Abcam, ab167429); anti-nesprin-1 rabbit monoclonal antibody clone EPR14196 (1:250; Abcam, ab192234); anti-nesprin-2 (1:300, kind gift from the Hodzic laboratory); anti-lamin A/C (1:1,000; Santa Cruz Biotechnology, sc376248); anti-lamin A/C mouse monoclonal antibody clone 4C11 (1:500; CST, 4777); phospho-H2A.X (1:1,000; MilliporeSigma, 05-636); anti-desmin goat polyclonal antibody (1:250; R&D Systems, AF3844); anti-desmin (1:1,000; PA-1151113); anti-MHY7 (1:250; DSHB, BA-D5); goat anti-rabbit IgG AF647 (1:1,000; Life Technologies, A27040); goat anti-mouse IgG AF488 (1:1,000; Life Technologies, A11001); colchicine (1 μM in DMSO, Sigma-Aldrich); isoproterenol (1 μM in DMSO); and blebbistatin (10 μM in DMSO, Cayman Chemical).

### Image acquisition

#### Live three-dimensional imaging of resting primary cardiomyocytes

For live three-dimensional super-resolution imaging, adult rat cardiomyocytes in culture media were loaded overnight with 0.1 µl ml^−1^ SPY650-DNA (Spirochrome) to label nuclei and 10 µM blebbistatin immediately before imaging to prevent motion artifacts. Airyscan SR (super resolution) *z* stacks were acquired with a Zeiss 880 Airyscan confocal microscope with ×63 oil 1.4 numerical aperture objective and 640-nm laser line in a glass-bottom dish. Raw images were processed with a joint deconvolution plugin (ZEN 3.5 Blue).

#### Live cGAS–tdTomato foci of resting primary cardiomyocytes

Live isolated cGAS–tdTomato cardiomyocytes were adhered to glass-bottom dishes using MyoTak (IonOptix) and loaded with 4 µM Hoechst and 10 µM blebbistatin immediately before imaging. Tile scan images were acquired at room temperature with a Zeiss 980 Airyscan confocal microscope equipped with Plan-Apochromat ×20 air 0.8 numerical aperture objective. Two channels (tdTomato and Hoechst), with 1-μm *z*-stack tile scans, were acquired, stitched and Airyscan processed using ZEN Black software. Nuclei from dead or severely deformed cells or with motion or stitching artifacts were excluded from analysis.

#### hiPSC-CM imaging

*z* stacks (10-µm range with voxel size of 0.03 × 0.035 × 0.5 µm) of individual nuclei were acquired on a Zeiss LSM 980 Airyscan 2 confocal microscope. A Plan-Apochromat ×63 oil 1.4 numerical aperture objective was used. Tile imaging was performed under ×4 zoom and ×2 bidirectional averaging. The SR-4Y Multiplexing acquisition mode was used for faster parallel pixel readout. Samples were excited with the 405-nm, 488-nm and 639-nm laser lines. For nuclei selection, the Hoechst channel was used to preview images, and 33.7 × 33.7-µm ROIs were defined to frame individual nuclei. To ensure examination of properly individualized hiPSC-CMs, ROIs containing more than one nucleus were not selected for imaging. Images were Airyscan processed using ZEN Black software.

#### Mouse tissue sections

Imaging was performed on two or more myocardial areas from each of at least three individual sections of every animal. Areas with clear striations corresponding to longitudinally sectioned cardiomyocytes were identified through the eyepiece. For the characterization of the cardiomyocyte cytoskeleton and chromatin protrusions, *z* stacks (6 × 1-μm slices, 6-μm range) were acquired on a Zeiss LSM 980 Airyscan 2 confocal microscope using a Plan-Apochromat ×63 oil 1.4 numerical aperture objective. Imaging was performed under ×1.7 zoom and ×2 bidirectional averaging, with 0.035 × 0.035-μm pixel size. Images were acquired in super-resolution acquisition mode at 2.0× Nyquist sampling. The 639-nm, 561-nm, 488-nm and 405-nm laser lines were used for imaging of desmin, α-tubulin, WGA and Hoechst, respectively. Alternatively, a ×20 air 0.8 numerical aperture objective was used to image immune cell markers, and 3-μm *z* stacks (four slices) were acquired. Images were acquired with ×1.7 digital zoom in confocal mode with no bidirectional averaging, resulting in 0.124 × 0.124-μm pixel size. The 639-nm, 488-nm and 405-nm laser lines were used for the imaging of CD45, CD68 and Hoechst, respectively. In all cases, images were Airyscan processed using ZEN Black software.

### Image analysis

Raw images were Airyscan processed with ZEN Black software and imported to Arivis V4D 4.0–4.1 for further analysis. Dedicated V4D analysis pipeline was generated to autosegment (Otsu) the nuclei from three-dimensional *z* stacks or two-dimensional maximum intensity projection (MIP) images and export nuclear morphology parameters, such as nuclear volume/area, length, width and aspect ratio.

#### Chromatin protrusions

To determine the percentage of nuclei with chromatin protrusions for each group in primary adult mouse cardiomyocytes or mouse tissue sections, three individuals were recruited to do a blinded analysis. In consistency with a recent report, nuclei were identified as bearers of chromatin protrusions when having both (1) an external outline deviating from the resting ovoid shape and (2) strong DNA condensation at the nuclear poles protruding from the expected nuclear ovoid shape, leading to nuclear deformation. In the case of the mouse tissue sections, only nuclei with a discernible outline within the *z* stack were included in the analysis. A stack of 819 (in the case of primary mouse cardiomyocytes) or 441 (for mice tissue sections) maximum intensity images of Hoechst-stained nuclei from all animals was given to each individual scorer to categorize as nuclei with or without a chromatin protrusion according to the abovementioned criteria. The average chromatin protrusions from each animal were defined from the percentage of chromatin protrusions scored by each blinded user. The average chromatin protrusions for each group were, in turn, derived from the average scores of individual animals of the corresponding group.

#### Perinuclear enrichment in isolated cardiomyocytes

Perinuclear MT or kinesin-1 enrichment was calculated on MIP images from 3 × 1-µm *z-*stack slices (total 3 µm) around the mid-plane of the nucleus. A perinuclear ring object was created from 0.5-µm dilation of the nuclear object and subtraction of the nucleus. Nuclear pole enrichment was calculated from manually traced 2-µm-wide objects for each of the long and short nuclear poles. To quantify the biphasic relationship between nuclear aspect ratio and perinuclear MT enrichment, a ‘LOESS’ smoothing algorithm was applied to the pooled data from all experimental groups (Fig. 5c) and revealed a deflection point at ‘MT cage enrichment’ = 1.9. This deflection point was further verified by fitting a piecewise linear regression, which indicated a negative correlation between nuclear aspect ratio and MT cage enrichment below 1.9 (slope = −2.1, *P* < 0.001). By contrast, the slope above this deflection point was flat (*P* > 0.05), indicating constant nuclear aspect ratio for MT cage enrichment ≥1.9.

#### cGAS–tdTomato foci

cGAS foci were calculated from tile MIP images with autosegmentation (Otsu) of the nuclei from the Hoechst channel and generation of 1-µm-perimeter perinuclear ring. Fixed intensity (>600 a.u. or >200 a.u. for the WT versus *Lmna* N195K cGAS–tdTomato models) and size (>0.8 µm^2^) thresholds for cGAS–tdTomato signal were used to segment the cGAS foci in the vicinity of the perinuclear ring. The segmented cGAS foci were used to quantify the percentage of cGAS-positive myocytes. To prevent contamination by cytosolic cGAS intensity, only foci touching the Hoechst signal and originating within the 1-µm-diameter perinuclear rings were included as readouts for nuclear ruptures.

#### γH2A.X foci

hiPSC-CM images were analyzed with custom Arivis V4D 4.1 pipeline to segment the Hoechst channel, using the Otsu method to outline the nuclei in three-dimensional *z* stacks. γH2A.X foci were segmented using fixed intensity (>2,000 a.u.) and volume (0.02–1,000 µm^3^) thresholds. Nuclear volume and mean nuclear intensity of γH2A.X signal were calculated using the nuclear segmentation. Puncta volume, integrated volume of all intranuclear puncta and number of puncta were calculated from the segmented γH2A.X images. The data were imported to MATLAB (MathWorks, R2022b) for pooling, and subsequent graphing and analysis was performed using Origin 2019 (OriginLab). The integrated volume of all γH2A.X foci within a nucleus was divided by the corresponding nuclear volume to calculate the fraction of the nuclear volume occupied by γH2A.X foci or foci fraction of volume coverage. The integrated volume of all γH2A.X foci within a nucleus was divided by the number of foci in that nucleus to calculate the mean γH2A.X foci volume. Data points with a value greater than 3 s.d. above the mean of the γH2A.X mean foci volume and/or of the number of γH2A.X foci were considered outliers and excluded from the analysis. These two parameters were used to identify outliers because they showed the highest variability among the measured parameters. Data were normalized to the mean of the non-targeted DMSO group for each experimental replicate.

#### Cardiomyocyte area coverage in tissue sections

Individual cardiomyocytes were segmented on MIP images from 4 × 1-µm *z*-stack slices (total 4 µm), using dedicated Arivis pipeline with watershed (membrane) algorithm on the WGA signal. The sum area of all segmented cardiomyocytes in a single image was divided by the total image area to calculate cardiomyocyte area coverage.

#### Cardiomyocyte nuclei and perinuclear cytoskeleton in tissue sections

Individual cardiomyocyte segments from cardiomyocyte area coverage analysis were superimposed with Hoechst channel to identify and select only cardiomyocytes with fully covered nuclei and generate a mask in the Hoechst channel with only the selected cardiomyocytes. The masked Hoechst channel was further background corrected and Otsu algorithm applied to automatically segment the cardiomyocyte nuclei. Furthermore, perinuclear ring segments were created from 0.5-µm dilation of the nuclear segments and subtraction of the nucleus. Similarly, cytoplasmic ring segments were created from 3-µm dilatation of the perinuclear segments. Nuclear morphology parameters were extracted from the nuclear segments and mean fluorescence desmin and α-tubulin intensities from the perinuclear and cytoplasmic segments.

#### Immune cell markers

CD45 and CD68 quantification in mouse heart tissue sections was performed using FIJI. Both channels were Gaussian blurred using a 0.3-μm sigma (radius), and MIPs were generated. Both the CD45 and CD68 channels were thresholded using a set intensity value. The percentage area coverage for each marker was calculated from the binary images. The average percent CD45 and CD68 coverage area for each animal was defined from the coverage values obtained from 5–6 myocardial regions of 246 µm × 246 µm.

### Statistics and reproducibility

Statistical analysis was performed using MATLAB, OriginPro (version 9 and 2018), GraphPad Prism (version 10.1.2) and R (version 4.0.2). Statistical tests and information on biological and technical replicates can be found in the figure legends. When applicable, individual nuclei or cells are indicated with open circles with statistical analysis performed on the pooled data. In addition, mean values of individual animal replicates are superimposed and labeled as closed triangles and connected by lines between the corresponding experimental conditions. For box and bar plots, the mean line is shown, with whiskers denoting s.e. or s.d. from the mean as indicated in each figure legend. Statistical tests for each comparison are denoted in the figure legends. Survival curves were generated using the Kaplan–Meier method, and differences in survival were tested with a Breslow test. Western blot images are representative of three independent replications.

### Computational model

The adult cardiomyocyte is rod shaped and often binucleated, with cylindrical symmetry that is largely preserved with *Lmna* mutations and LINC complex perturbations. For simplicity, we leverage this symmetry and consider an axisymmetric model covering only a quarter of the cardiomyocyte (Fig. 8a). Using COMSOL multi-physics software, we simulate nuclear morphological changes due to the mutation and LINC complex disruption. Figure 8a illustrates a typical finite element mesh used for these simulations. According to this figure, the model comprises three main parts: (1) myofibrils (cytoplasm), (2) nucleus and (3) perinuclear MT cage. The cell radius, cell length and nuclear radius are set to 35 µm, 100 µm and 4 µm, respectively. The NE thickness (including the lamina) is set to 0.2 µm, and the MT cage long and short axis lengths are set to 8 µm and 4.4 µm, respectively.

#### Myofibrils

Adult cardiomyocytes are predominantly filled with myofibrils, encapsulated by the surrounding sarcolemma. Myofibrils are long contractile fibers that comprise primarily interdigitating actin and myosin filaments, which are precisely held together by titin proteins^49^. Experimental observations show that, during diastole, actomyosin interaction is not fully off, and, thus, there is an active contraction in resting cardiomyocytes^27^. This contraction encounters resistance from restoring forces mediated by titin protein^27^ and geometric constraints imposed by the myocardium microenvironment^29^. Our simulations (discussed later) demonstrate that this diastolic contraction laterally compresses central nuclei, causing them to elongate in the axial direction. This nuclear elongation, in turn, compresses the surrounding MT cage, generating pushing elastic forces in the perinuclear MT cage. Consequently, a complex pre-stress field exists in the cardiomyocyte that governs the nuclear morphology changes. The existence of this resting stress field is supported by the observation that the elongated cardiomyocyte nuclei become round upon isolation^28^. To induce this stress field in our model, we note that available experiments^50^ show that myofibrils tend to assemble in the direction of maximum principal stress^51^. This implies that the myofibrillar organization during maturation is mainly controlled by stress-activated signaling pathways (such as calcium pathway)^29^. Therefore, we employ our previously developed chemo-mechanical model^52^ to capture this stress-dependent assembly of the myofibrils. To this end, we first hypothesize an (imaginary) stress-free configuration for the cardiomyocyte, wherein the nuclei are round and actomyosin fibers (myofibrils) are randomly distributed and not yet assembled (Fig. 8a). We further assume the following relation among the myofibrillar pre-stress field (${\sigma }_{{ij}}^{{\rm{mf}}}$), the rest contractility (shown by tensor *ρ*_*ij*_) and the corresponding strain field (*ε*_*ij*_). Subscript *k* in *ε*_*k*__*k*_ refers to the trace of the strain tensor (that is, the sum of its diagonal components (volumetric strain)), and *δ* is the Kronecker delta, which is 1 when *i* = *j* and 0 otherwise (see ref. ^52^ for more details):

E1 $${\sigma }_{{ij}}^{{\rm{mf}}}=\bar{K}{\varepsilon }_{{kk}}{\delta }_{{ij}}+2\bar{\mu }\left({\varepsilon }_{{ij}}-\frac{1}{3}{\varepsilon }_{{kk}}{\delta }_{{ij}}\right)+{\bar{\rho }}_{0}{\delta }_{{ij}},$$

E2 $${\rho }_{{ij}}={\bar{\rho }}_{0}{\delta }_{{ij}}+{\bar{K}}_{\rho }{\varepsilon }_{{kk}}{\delta }_{{ij}}+2{\bar{\mu }}_{\rho }\left({\varepsilon }_{{ij}}-\frac{1}{3}{\varepsilon }_{{kk}}{\delta }_{{ij}}\right),$$

where we have:

E3 $${\bar{\rho }}_{0}=\frac{\beta {\rho }_{0}}{\beta -{\alpha }_{\rm{v}}},\,3\bar{K}=\frac{3K\beta -1}{\beta -{\alpha }_{\rm{v}}},\,2\bar{\mu }=\frac{2\mu \beta -1}{\beta -{\alpha }_{\rm{v}}},\,{3\bar{K}}_{\rho }=\frac{3K{\alpha }_{v}-1}{\beta -{\alpha }_{\rm{v}}},\,2{\bar{\mu }}_{\rho }=\frac{2\mu {\alpha }_{\rm{v}}-1}{\beta -{\alpha }_{\rm{v}}},$$

in which *β* is the chemical stiffness (set to 2.77 kPa^−1^ (ref. ^52^)); *α*_v_ is the volumetric chemo-mechanical feedback parameter (set to 2.3 kPa^−1^ (ref. ^52^)); and $K=E/(3(1-2\nu ))$ and $\mu =E/(2\left(1+\nu \right))$ are the bulk and shear moduli with *E* and *ν* as the elastic modulus and Poisson’s ratio, respectively. For both the cytoplasm and MT cage, elastic moduli *E* is set to 1.2 kPa, with Poisson’s ratio *ν* of 0.3 (ref. ^20^). Furthermore, *ρ*_0_ is the initial contractility, which is 0 in the stress-free configuration.

By increasing the initial contractility *ρ*_0_ to 1.2 kPa (estimated; Fig. 8b), we then simulate the assembly of myofibrils, resulting in a tendency for the cardiomyocyte to shrink. However, this cell shrinkage is resisted, primarily in the longitudinal direction, by titin proteins and the geometric constraints of the cardiomyocyte microenvironment^53^, as mentioned earlier. Accordingly, we restrict the longitudinal displacement of the cell at its two ends. Therefore, radial contraction occurs in the cell while its length remains fixed (Supplementary Video 3). This constrained contraction gives rise to the generation of an anisotropic stress field inside the cytoplasm, as illustrated in Fig. 8c and Extended Data Fig. 10a. As a result of this anisotropic stress field, the contractility tensor *ρ*_*ij*_ will be no longer isotropic (equation (E2)) because stress-activated signaling pathways promote formation of myofibrils in the direction of maximum principal stress^51,54^. Extended Data Fig. 10b illustrates the obtained directions for the maximum principal stress (maximum tensile stress) field inside the cytoplasm. Notably, there is a great match between these directions and the directions of myofibrils in the physiological conditions.

#### Nucleus

The nucleus is primarily composed of two key mechanical components: chromatin and the NE with its underlying lamina. The NE includes nuclear membranes and nuclear pore complexes, and chromatin serves as the primary building block of the nucleoplasm. Experimental observations^55^ indicate that chromatin plays a crucial role in resisting small nuclear deformations, whereas NE and its underlying lamina predominate in scenarios involving large nuclear deformations. These experiments further suggest that the lamina network, especially lamins A and C, exhibits substantial strain stiffening, whereas chromatin remains linear even at large deformations. Consequently, we model the nucleoplasm (chromatin) as a linear elastic material with a Young’s modulus of 150 Pa (ref. ^54^) and the NE and its underlying lamina as a neo-Hookean hyperelastic layer with an elastic modulus of 15 kPa (refs. ^56,57^). This hyperelastic material model captures the strain stiffening of the NE and its underlying lamina upon large deformation. Furthermore, our in vivo observations demonstrate that relative nuclear volume changes due to lamin mutation and LINC complex disruption are less than 10%. Based on these findings, we further consider the nucleoplasm (chromatin) to be nearly incompressible with *ν* = 0.49, whereas the NE and its underlying lamina are modeled as a fully incompressible hyperelastic layer with *ν* = 0.5, similar to the approach in ref. ^56^.

#### Perinuclear MT cage

In adult cardiomyocytes, MT organizing centers are predominantly associated with the NE^58^. MTs anchor to the NE through the LINC complex, particularly nesprin-1 proteins and kinesin motors^16^, forming a dense and active cage around the nucleus. Consequently, MTs can exert active pushing forces on the nucleus through their polymerization and recruitment of the kinesin motors. This is supported by our super-resolution images (Fig. 5d), which reveal a wavy form of MTs around the nucleus, indicating that these rod-shaped fibers are under compression. To account for these active forces and for simplicity, we introduce an isotropic and homogenous compressive stress field with magnitude *σ*_MT_ to the perinuclear MT cage (equation (E4)). We increase this external stress from 0 in the stress-free configuration to 500 Pa when inducing the pre-stress field under WT (physiological) conditions (Fig. 8b). Furthermore, considering the observed MT wavelengths, typically between 2 μm and 3 μm (Fig. 5), the compressive force experienced by the MTs is estimated to be 100 pN or more^59^. This force is 10 times larger than the force of MT polymerization and 14–15 times larger than the force applied by kinesin motors^60^. Consequently, we assume that MTs are also under passive elastic compression in adult cardiomyocytes. Accordingly, we model the MT cage as an (active) linear elastic ellipsoid in the stress-free configuration. The constitutive equation for this cage can be written as:

E4 $${\sigma }_{{ij}}^{{\rm{MT}}}={K}_{{\rm{MT}}}{\varepsilon }_{{kk}}{\delta }_{{ij}}+2{\mu }_{{\rm{MT}}}\left({\varepsilon }_{{ij}}-\frac{1}{3}{\varepsilon }_{{kk}}{\delta }_{{ij}}\right)-{\sigma }_{{\rm{MT}}}{\delta }_{{ij}},$$

where ${K}_{{MT}}$*K*_MT_ and ${\mu }_{{MT}}$*μ*_MT_ are the bulk and shear moduli of the MT cage, respectively. Figure 8c and Extended Data Fig. 10e show the obtained stress field in the cage. According to these figures, the MT cage imposes maximum compressions on the long and short tips of the nucleus in the longitudinal (*z* direction) and radial (*r* direction) directions, respectively. This is consistent with our current observations that nuclear ruptures mostly occur at the long tips and also with our recent experimental findings^3^ that MTs indent into desmin knockdown nuclei mostly around the short sides and in the radial direction.

### Reporting summary

Further information on research design is available in the Nature Portfolio Reporting Summary linked to this article.

## Supplementary information

Reporting Summary

Supplementary Video 1 Electrically stimulated (1 Hz) adult rat cardiomyocyte labeled with SiR-actin and Hoechst to visualize sarcomeres and DNA, respectively.

Supplementary Video 2 Electrically stimulated (1 Hz) adult rat cardiomyocyte labeled with SPY-555 tubulin and Hoechst demonstrating MT cage buckling during contraction.

Supplementary Video 3 Cardiomyocyte model simulation following assembly of myofibrils (increased myofibril prestress with constant cytoplasm length) demonstrating development of anisotropic stress field within the cell with overall cellular radial compression and nuclear elongation.

## Source data

Source Data Fig. 7 Unprocessed western blot.

Source Data Extended Data Fig./Table 5 Unprocessed western blot.

Source Data Extended Data Fig./Table 7 Unprocessed western blot.

## Data Availability

Data supporting the findings of this study are included in the article, extended data and source data files.

## Code availability

Custom code used for the computational model simulations in this study is available on GitHub at https://github.com/MohammadDehghany/Nature-Cardiovascular-Research-COMSOL-Model.
