# Supplementary material for: Microtubule forces drive nuclear damage in LMNA cardiomyopathy
Source: Nat Cardiovasc Res. 2025 Oct 10;4(11):1501–20. doi: 10.1038/s44161-025-00727-w (PMC12611788; doi:10.1038/s44161-025-00727-w)
Supplement: Supplementary file 1 — Reporting Summary [file 44161_2025_727_MOESM1_ESM.pdf]

nature portfolio

Corresponding author(s): Benjamin L. Prosser

Last updated by author(s): Aug 18, 2025

Reporting Summary

Nature Portfolio wishes to improve the reproducibility of the work that we publish. This form provides structure for consistency and transparency in reporting. For further information on Nature Portfolio policies, see our Editorial Policies and the Editorial Policy Checklist.

Statistics

For all statistical analyses, confirm that the following items are present in the figure legend, table legend, main text, or Methods section.

n/a	Confirmed	
☐	☒	The exact sample size (*n*) for each experimental group/condition, given as a discrete number and unit of measurement
☐	☒	A statement on whether measurements were taken from distinct samples or whether the same sample was measured repeatedly
☐	☒	The statistical test(s) used AND whether they are one- or two-sided<br>*Only common tests should be described solely by name; describe more complex techniques in the Methods section.*
☐	☒	A description of all covariates tested
☐	☒	A description of any assumptions or corrections, such as tests of normality and adjustment for multiple comparisons
☐	☒	A full description of the statistical parameters including central tendency (e.g. means) or other basic estimates (e.g. regression coefficient) AND variation (e.g. standard deviation) or associated estimates of uncertainty (e.g. confidence intervals)
☐	☒	For null hypothesis testing, the test statistic (e.g. *F*, *t*, *r*) with confidence intervals, effect sizes, degrees of freedom and *P* value noted<br>*Give P values as exact values whenever suitable.*
☒	☐	For Bayesian analysis, information on the choice of priors and Markov chain Monte Carlo settings
☒	☐	For hierarchical and complex designs, identification of the appropriate level for tests and full reporting of outcomes
☒	☐	Estimates of effect sizes (e.g. Cohen's *d*, Pearson's *r*), indicating how they were calculated

Our web collection on statistics for biologists contains articles on many of the points above.

Software and code

Policy information about availability of computer code

Data collection	All software used for data collection has been described in the methods section. Specifically: IonWizard by IonOptix was used for sarcomere length measurements. Zenn Black by Zeiss was used for all image acquisition. COMSOL was used for computational modeling. ImageQuant LAS 500 was used for WB imaging. Vevo 2100 imaging system by VisualSonics was used for echocardiography.
Data analysis	All software used for data analysis has been described in the methods section. Specifically: IonWizard by IonOptix was used for analyzing sarcomere length measurements. Zenn Black by Zeiss was used for image Airyscan processing and Arivis V4D 4.0-4.1 was used for image analysis. Matlab R2022b by Mathworks was used for further combined analysis of the strain coupling data. Origin 2019 by OriginLab was used for graphing and statistical analysis. FIJI built into the ImageQuant LAS 500 was used for WB analysis. AutoLV Analysis Software by VisualSonics was used for echocardiography analysis.

For manuscripts utilizing custom algorithms or software that are central to the research but not yet described in published literature, software must be made available to editors and reviewers. We strongly encourage code deposition in a community repository (e.g. GitHub). See the Nature Portfolio guidelines for submitting code & software for further information.

Data

Policy information about availability of data

All manuscripts must include a data availability statement. This statement should provide the following information, where applicable:

- Accession codes, unique identifiers, or web links for publicly available datasets
- A description of any restrictions on data availability
- For clinical datasets or third party data, please ensure that the statement adheres to our policy

The data that support the plots within this paper and other findings of this study are available from the corresponding author upon reasonable request.

Research involving human participants, their data, or biological material

Policy information about studies with human participants or human data. See also policy information about sex, gender (identity/presentation), and sexual orientation and race, ethnicity and racism.

Reporting on sex and gender	N/A
Reporting on race, ethnicity, or other socially relevant groupings	N/A
Population characteristics	N/A
Recruitment	N/A
Ethics oversight	N/A

Note that full information on the approval of the study protocol must also be provided in the manuscript.

Field-specific reporting

Please select the one below that is the best fit for your research. If you are not sure, read the appropriate sections before making your selection.

☒ Life sciences ☐ Behavioural & social sciences ☐ Ecological, evolutionary & environmental sciences

For a reference copy of the document with all sections, see nature.com/documents/nr-reporting-summary-flat.pdf

Life sciences study design

All studies must disclose on these points even when the disclosure is negative.

Sample size	Sample size was determined based on author's previous experience as well as similar experiments in the published literature to inform on predicted effect sizes and subsequent power analyses. The number of replicates and number of cells used for each experiment are provided in the manuscript.
Data exclusions	For strain coupling analysis, recordings from individual cardiomyocytes with sarcomere length strain lower than 10% or baseline sarcomere length below 1.7 µm were excluded from analysis. No animal replicates were excluded from analysis.
Replication	All experiments were reproduced and the number of biological replications for each experiment is listed in the manuscript
Randomization	For in-vivo mice experiments mice of the desired genotypes were randomized for treatment. For in-vitro experiments, treatment groups were randomly allocated to the different cell containing wells.
Blinding	Scoring of chromatin protrusion was done by blinded observer analysis as described in the methods section. Unbiased analytical approaches were used to limit investigator bias. Echocardiography assessments were done blind to genotype/treatment group.

Reporting for specific materials, systems and methods

We require information from authors about some types of materials, experimental systems and methods used in many studies. Here, indicate whether each material, system or method listed is relevant to your study. If you are not sure if a list item applies to your research, read the appropriate section before selecting a response.

Materials & experimental systems

n/a	Involved in the study
☐	☒ Antibodies
☐	☒ Eukaryotic cell lines
☒	☐ Palaeontology and archaeology
☐	☒ Animals and other organisms
☒	☐ Clinical data
☒	☐ Dual use research of concern
☒	☐ Plants

Methods

n/a	Involved in the study
☒	☐ ChIP-seq
☒	☐ Flow cytometry
☒	☐ MRI-based neuroimaging

Antibodies

Antibodies used

Anti-alpha Tubulin mouse monoclonal antibody, clone DM1A (1:500, abcam ab264493);
Anti-alpha Tubulin mouse monoclonal antibody (1:500, Sigma Aldrich T5168, clone B-5-1-2);
Anti-KIF5B rabbit monoclonal antibody clone EPR10276(B) (1:500, abcam ab167429);
Anti-Nesprin1 rabbit monoclonal antibody clone EPR14196 (1:250, abcam ab192234);
Anti-Lamin A/C mouse monoclonal antibody clone 4C11 (1:500, Cell Signaling #4777);
Anti-lamin A/C (1:1000, sc-376248);
Anti-phospho-H2A.X mouse monoclonal antibody clone JBW301 (1:1000, 05-636, Millipore Sigma);
Anti-Nesprin-2 (1:300, Hodzic lab);
Anti-Desmin goat polyclonal antibody (1:250, R&D Systems AF3844);
Anti-Desmin (1:1000, PA-1151113);
Anti-MHY7 (1:250, DSHB BA-D5);
Anti-H3 (CST #44995);
Anti-GAPDH (CST#2118S)
Goat polyclonal anti-CD45 (R&D Systems AF114);
Rabbit monoclonal anti-CD68 (Cell Signaling Technology 97778);
Goat anti-rabbit IgG AF 647 (Life Technologies, A27040);
Goat anti-mouse IgG AF488 (1:1000, Life Technologies, A11001);
Alexa Fluor 488 conjugated WGA (Invitrogen, W11261)

Validation

All antibodies except nesprin-2 came from commercial vendors and were validated by the manufacturers on their official website. Nesprin-2 antibody was validated by the source lab (Didier Hodzic)

Eukaryotic cell lines

Policy information about cell lines and Sex and Gender in Research

Cell line source(s)

NCardia vCardiomyocytes: https://www.ncardia.com/discovery-services/modeling/ncyte-vcardiomyocytes.
Cat #: Nc-C-BRCM
Lot #: 6280620G20

Authentication

Cell lines were not authenticated

Mycoplasma contamination

Negative for mycoplasma

Commonly misidentified lines
(See ICLAC register)

N/A

Animals and other research organisms

Policy information about studies involving animals; ARRIVE guidelines recommended for reporting animal research, and Sex and Gender in Research

Laboratory animals

8-12 week old, male Sprague Dawley rats were used for the isolation of primary adult cardiomyocytes. 8-9 week old, C57BL/6 background male and female mice were used for survival, cardiac function studies and isolation of primary adult cardiomyocytes, with the following genetic background:
1. LmnaN195K/N195K and littermate controls
2. αMHC Cre+/- KASH+/- after tamoxifen or vehicle injections at 3 weeks
3. αMHC Cre+/- KASH+/- LmnaN195K/N195K after tamoxifen or vehicle injections at 3 weeks
4. LmnaN195K/N195K mice expressing cGAS-tdTomato
5. αMHC- Cre+/- Lmna+/- mice after tamoxifen or vehicle injections at 10 weeks, with colchicine or PBS control as treatment injections

Wild animals

The study did not involve wild animals.

Reporting on sex

Survival and echocardiography data are presented separately for males and females in Lmna N195K and Lmna cKO mouse models.

Field-collected samples	The study did not involve field-collected samples.
Ethics oversight	Animal care and use procedures were performed in accordance with the standards set forth by the University of Pennsylvania Institutional Animal Care and Use Committee and the Guide for the Care and Use of Laboratory Animals published by the US National Institutes of Health. Protocols were approved by the University of Pennsylvania Institutional Animal Care and Use Committee. All animals provided by the Lammerding Lab at Cornell were bred and maintained according to relevant guidelines and ethical regulations approved by the Cornell University Institutional Animal Care and Use Committee, protocol 2011-0099.

Note that full information on the approval of the study protocol must also be provided in the manuscript.

Plants

Seed stocks	N/A
Novel plant genotypes	N/A
Authentication	N/A
