# Supplementary figures and images for: Microtubule forces drive nuclear damage in LMNA cardiomyopathy
Source: Nat Cardiovasc Res. 2025 Oct 10;4(11):1501–20. doi: 10.1038/s44161-025-00727-w (PMC12611788; doi:10.1038/s44161-025-00727-w)

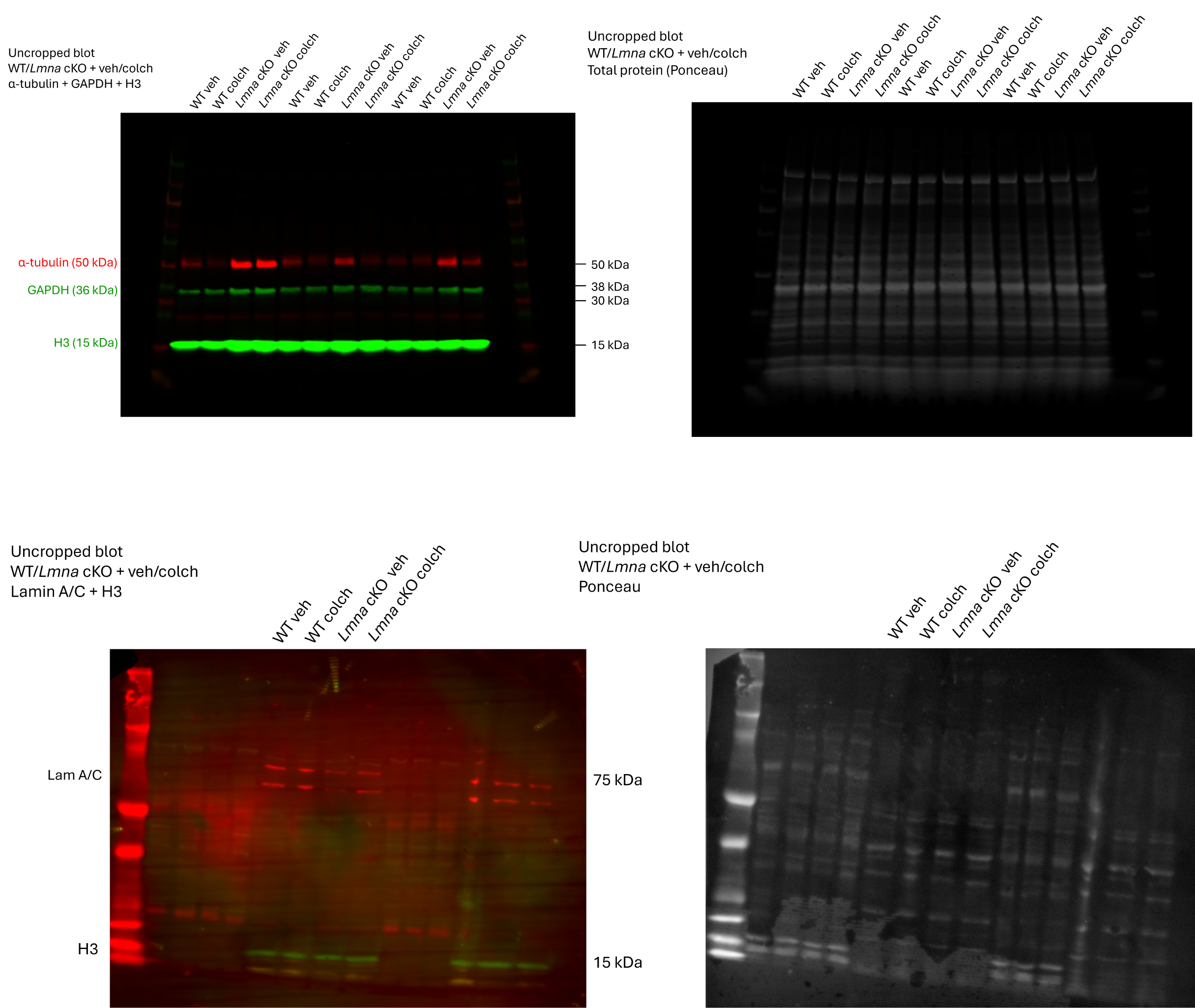

Supplement: Supplementary file 5 — Unprocessed western blot. [file 44161_2025_727_MOESM5_ESM.tif]

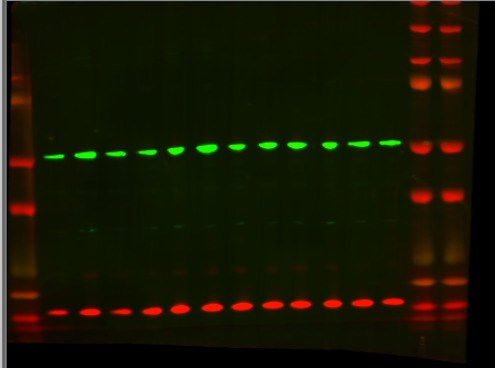

Supplement: Supplementary file 6 — Unprocessed western blot. [file 44161_2025_727_MOESM6_ESM.tif]

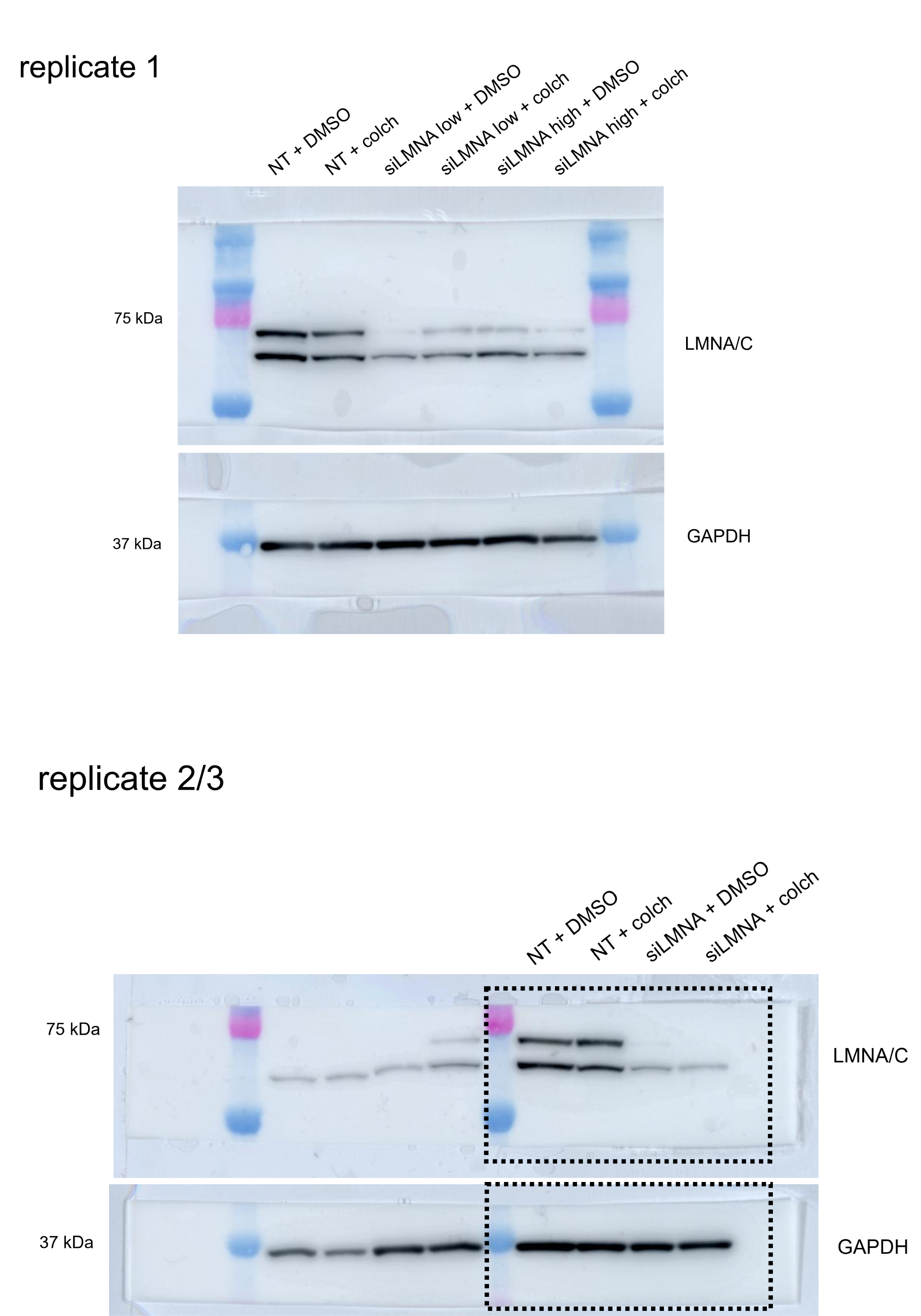

Supplement: Supplementary file 7 — Unprocessed western blot. [file 44161_2025_727_MOESM7_ESM.tif]
